# Structural basis of water-mediated *cis* Watson–Crick/Hoogsteen base-pair formation in non-CpG methylation

**DOI:** 10.1093/nar/gkae594

**Published:** 2024-07-11

**Authors:** Shan-Meng Lin, Hsiang-Ti Huang, Pei-Ju Fang, Chi-Fon Chang, Roshan Satange, Chung-ke Chang, Shan-Ho Chou, Stephen Neidle, Ming-Hon Hou

**Affiliations:** Graduate Institute of Genomics and Bioinformatics, National Chung Hsing University, Taichung 402, Taiwan; Graduate Institute of Genomics and Bioinformatics, National Chung Hsing University, Taichung 402, Taiwan; Genomics Research Center, Academia Sinica, Taipei 115, Taiwan; Genomics Research Center, Academia Sinica, Taipei 115, Taiwan; Graduate Institute of Genomics and Bioinformatics, National Chung Hsing University, Taichung 402, Taiwan; Taiwan Biobank, Institute of Biomedical Sciences, Academia Sinica, Taipei 115, Taiwan; Institute of Biochemistry, National Chung Hsing University, Taichung 402, Taiwan; School of Pharmacy, University College London, London WC1N 1AX, UK; Graduate Institute of Genomics and Bioinformatics, National Chung Hsing University, Taichung 402, Taiwan; Doctoral Program in Medical Biotechnology, National Chung Hsing University, Taichung 402, Taiwan; Graduate Institute of Biotechnology, National Chung Hsing University, Taichung 402, Taiwan; Department of Life Sciences, National Chung Hsing University, Taichung 402, Taiwan; Biotechnology Center, National Chung Hsing University, Taichung 402, Taiwan

## Abstract

Non-CpG methylation is associated with several cellular processes, especially neuronal development and cancer, while its effect on DNA structure remains unclear. We have determined the crystal structures of DNA duplexes containing -CGCCG- regions as CCG repeat motifs that comprise a non-CpG site with or without cytosine methylation. Crystal structure analyses have revealed that the _m_C:G base-pair can simultaneously form two alternative conformations arising from non-CpG methylation, including a unique water-mediated *cis* Watson–Crick/Hoogsteen, (w)cWH, and Watson–Crick (WC) geometries, with partial occupancies of 0.1 and 0.9, respectively. NMR studies showed that an alternative conformation of methylated _m_C:G base-pair at non-CpG step exhibits characteristics of cWH with a *syn*-guanosine conformation in solution. DNA duplexes complexed with the DNA binding drug echinomycin result in increased occupancy of the (w)cWH geometry in the methylated base-pair (from 0.1 to 0.3). Our structural results demonstrated that cytosine methylation at a non-CpG step leads to an *anti*→*syn*transition of its complementary guanosine residue toward the (w)cWH geometry as a partial population of WC, in both drug-bound and naked _m_C:G base pairs. This particular geometry is specific to non-CpG methylated dinucleotide sites in B-form DNA. Overall, the current study provides new insights into DNA conformation during epigenetic regulation.

## Graphical Abstract

**Graphical Abstract:**
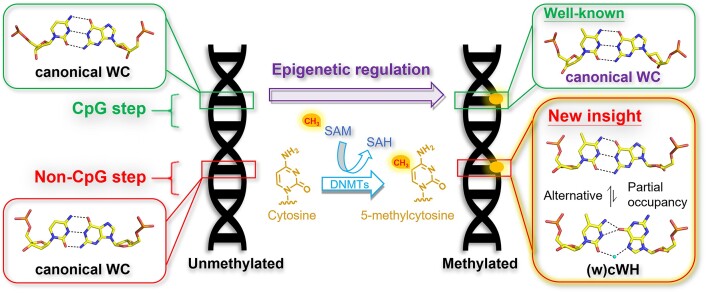

## Introduction

DNA methylation is an important epigenetic modification mechanism that enables the regulation of gene expression without altering its DNA sequence (1–5). In this process a methyl group is transferred to the pyrimidine ring of a cytosine at the fifth carbon position, forming a stable 5-methyl cytosine (5_m_C) residue. The reaction is catalyzed by specific enzymes termed DNA methyltransferases and is tightly regulated. Aberrant methylation is a well-established molecular lesion in cancer (6–15) and has also been associated with other diseases, including neurodegeneration (2,8,16,17). There are two types of DNA methylation: CpG methylation occurs at cytosine residues immediately preceding a guanine residue and is often found in promoter regions (18,19). The process is catalyzed by the DNMT1 methyltransferase. On the other hand, methylation of cytosines immediately preceding a non-guanine residue is known as non-CpG methylation (15,20–27). Non-CpG methylation is often observed within genes and is catalyzed by another set of DNA methyltransferases, DNMT3a and DNMT3b (28–30). Other proteins, including the Enhancer of Zeste Homolog 2 (EZH2) and the methyl-CpG binding protein 2 (MeCP2), are also involved in the regulation of non-CpG methylation (31–35). Interestingly, non-CpG methylation in the human genome has been shown to correlate spatially with CpG methylation, suggesting a functional connection between the two types of DNA methylation (21).

Methylation of DNA regulates gene expression via two possible mechanisms: one is through the direct effect of the methyl moiety, which may physically interact with DNA-binding proteins and either enhance or inhibit their recruitment. The other is by exerting an indirect effect on the biophysical properties of DNA, such as changing its conformation or rigidity (36–43). Studying the effects of DNA methylation on DNA structure is thus crucial for understanding gene regulation. However, most structural studies have focused on the various aspects of CpG methylation. For non-CpG methylation, studies have focused on the complexes formed between the methylated DNA and its binding protein (44–50), but relatively little is known about the structural effects of non-CpG methylation on DNA itself.

In DNA structure, Watson–Crick (WC) base pairs, characterized by their specific hydrogen bonding patterns between complementary nucleobases (A-T, G-C), play a major role in stabilizing the DNA double helix, which is essential for the storage and replication of genetic information (51). Additionally, nucleotide base pairs can also adopt a variety of geometries under specific conditions. These base pairs, commonly referred to non-canonical base pairs (52), have their distinctive hydrogen bonding patterns differing from those observed in canonical WC and have been well-defined (53). Non-canonical base pairs can participate in those biological processes that cannot be achieved through WC pairing alone, thereby broadening the structural and functional diversity of the DNA duplex (54–56). For example, the Hoogsteen (HG) base pair (57), containing a purine nucleotide undergoes a 180° rotation around the glycosidic bond (58), and can play a pivotal role in the specific binding of some proteins to DNA (59). Accordingly, it has been reported to be involved in a range of biological processes (60,61), such as DNA replication (61,62), DNA repair (60), as well as DNA recognition by the tumor suppressor p53 (63–65). On the other hand, recent NMR relaxation experiments on native DNA have revealed that the predominant WC base pairing is in dynamic equilibrium with a transient HG (66–70), and this WC-to-HG transition may be crucial for protein recognition (61,67,71). Understanding the geometric differences of DNA base pairs under specific biological conditions is thus of considerable potential significance, as it may provide insights into the mechanisms governing genetic processes beyond the primary information encoded within the nucleotide sequence.

To investigate the structural effects of non-CpG methylation on the DNA structure, we have determined the crystal structures of the DNA duplexes d(ACG_m_**C**CGT/ACG**G**CGT) (named as _m_C:G pairing structure)and d(ACG**C**CGT/ACG**G**CGT) (named as C:G pairing structure), which represent methylated and unmethylated states, respectively. These crystal structures have revealed that cytosine methylation at a non-CpG site flanking two CpGs resulted in polymorphic conformations of the central _m_C:G base pair, including the unexpected formation of a novel water-mediated *cis* Watson–Crick/Hoogsteen, (w)cWH, base pair arrangement at partial occupancy, which is specific to methylation at a non-CpG site in B-form DNA. Consistent with these structural findings, parallel NMR studies have indicated that non-CpG methylated _m_C:G base-pair can adopt two alternative conformations in solution, one of which exhibited characteristics of Hoogsteen geometry. Additionally, we applied a DNA binding drug, echinomycin (ECHI), which is expected to stabilize (w)cWH near the drug intercalating site, to manipulate the occurrence of (w)cWH. An increased occupancy of (w)cWH geometry (from 0.1 to 0.3) was observed in the crystal structure of the _m_C:G-ECHI complex. This suggests that a conserved base pair geometry could also form with a non-CpG methylated _m_C:G base pair upon drug binding. These results highlight the significance of the unique (w)cWH geometry and provide the first insight into the effects of non-CpG methylation on the structure of a DNA duplex.

## Materials and methods

### Oligonucleotide sample preparation

All chemical reagents were purchased from Sigma Chemical Co. (St. Louis, MO, USA). DNA oligonucleotides were purchased from MD Bio (Taipei, Taiwan). DNA and echinomycin stock solutions were prepared in water and DMSO, respectively. The DNA oligonucleotide and echinomycin concentrations were determined by measuring the optical density at 260 and 440 nm, respectively, using a JASCO V-630 spectrophotometer (JASCO International Co. Ltd., Tokyo, Japan). The C:G [d(ACG**C**CGT/ACG**G**CGT)], _m_C:G [d(ACG**_m_C**CGT/ACG**G**CGT)] and C:I [d(ACG**C**CGT/ACG**I**CGT)] pairing duplexes were prepared in water via heating at 95°C for 5 min followed by cooling on ice for 30 min to allow duplex formation.

### Crystallization

To obtain the unliganded C:G pairing crystals, 0.5 mM of the C:G pairing duplex was mixed in a solution containing 50 mM MES (pH = 6.5), 1 mM spermine HCl, 20% PEG400, and equilibrated against 500 μl of 30% PEG 400 at 20°C for 1 week. To obtain the unliganded _m_C:G pairing crystals, 0.45 mM of _m_CG duplex was mixed in a solution containing 50 mM MES (pH = 6.5), 1 mM spermine HCl, 20% PEG400, and equilibrated against 500 μl of 30% PEG 400 at 20°C for 1 week. To obtain the unliganded C:I pairing crystals, 0.125 mM of the C:I pairing duplex was mixed in a solution containing 30 mM MES (pH = 6.5), 1 mM spermine HCl, 8% PEG400, 1.5 mM MgCl_2_, and equilibrated against 500 μl of 50% PEG 400 at 20°C for 1 week. To prepare the DNA:ECHI complexes, 0.125 mM DNA duplex was pre-incubated with 0.275 mM ECHI at 4°C for 72 h. For the C:G-ECHI complex, crystals were grown for three weeks in a 5 μl drop containing 20 mM MES (pH = 6), 10 mM spermine HCl, 1% PEG 200, 10 mM MnCl_2_, equilibrated against 500 μl of 30% PEG 200 at 20°C. For the _m_C:G-ECHI complex, crystals were grown for three weeks in a 5 μl drop containing 20 mM MES (pH = 6), 5 mM spermine HCl, 1.2% PEG 200, 10 mM MnCl_2_, equilibrated against 500 μl of 30% PEG 200 at 20°C.

### X-ray data collection, phasing, and structure refinement

X-ray diffraction data of unliganded C:G, _m_C:G, C:I pairing crystals and C:G-ECHI, _m_C:G-ECHI complex crystals were collected at the National Synchrotron Radiation Research Center, Hsinchu, Taiwan. The diffraction data for the unliganded C:G, C:I pairing crystals and the C:G-ECHI complex crystals were collected at beamline BL15A1 and recorded using a Rayonix MX300HE CCD area detector. Data for unliganded _m_C:G pairing crystals and _m_C:G-ECHI complex crystals were collected on beamline TPS 05A with a Rayonix MX300HS CCD detector. Diffraction data integration and reduction were conducted using the HKL-2000 package (72). The phases of these structures were solved using phaser-MR in PHENIX v1.17.1. The phases of the unliganded duplexes and DNA:ECHI complexes were solved using the partial structure of the DNA duplex (PDB ID:6JV5) (73) and ECHI-d(ACGTCGT)_2_ (PDB ID:5YTZ) (74), respectively. Structural refinements were performed using the PHENIX package (v1.17.1.). In brief, all crystal structures were refined using the PHENIX package through three cycles of refinement in phenix.refine with default settings, along with the inclusion of hydrogen bond restraints. The refinement programs generated 2mF_o_-DF_c_ and mF_o_-DF_c_ difference maps, where ‘F_o_ and F_c_’ are the experimentally observed and calculated amplitudes, respectively. ‘m’ denotes the figure of merit, and ‘D’ represents the Sigma-A weighting factor (75). The 2mF_o_-DF_c_ map indicates the area where the model is most expected to be located. The mF_o_-DF_c_ map, depicted with positive and negative contours, reveals the areas where atoms are lacking in the current model or where atoms exist in the model but are not present in the crystal, respectively (76). The refinement of (w)cWH base pairs were completed followed the subsequent procedure: (i) _m_C:G base pairs in the _m_C:G pairing duplex and _m_C:G-ECHI complex structures were initially modeled with a single guanosine in either the *anti* or *syn* conformation, and refined using the aforementioned phenix.refine routine. Following refinement, ambiguous maps were observed at the methylated _m_C:G base pairs in one of the four asymmetric units of the unliganded _m_C:G pairing structure and in the _m_C:G-ECHI structures (see Supplementary Figure S13A, B and E, F). (ii) An alternative guanosine residue was modeled into _m_C:G pairs, and a second round of refinement was carried out with the same phenix.refine routine. (iii) Based on mF_o_-DF_c_difference maps depicted with positive contours, a water molecule was modelled into _m_C:G pair and further refined using the same phenix.refine routine (see Supplementary Figure S13C and G). The refinement was ultimately optimized through B-factor refinement and group occupancy refinement (see Supplementary Figure S13D and H)

### MD simulation protocol

The MD simulation was conducted based on the protocol modified from previous studies (77). Briefly, crystal structures of the representative duplexes for unliganded C:G, _m_C:G, and C:I pairing structures were used for MD simulations, and Discovery Studio software v19.1.0.18287 with the CHARMm force field was applied for the simulations (78). After a 1000 step smart-minimizer, the models were immersed in a water box built with a modified TIP3P system (79). The total charge on each system was neutralized by the random addition of sodium ions. Then a 5000-step Steepest Descent (SD) and 10 000-step conjugate gradient minimization was run, followed by heating from 50 to 300 K for 20 ps with position constraints on the DNA duplexes. Consequently a 40 ps equilibration was performed with position constraints on the DNA duplexes. The production run was performed by using an NPT ensemble with periodic boundary conditions. The terminal bases of the DNA duplex were constrained using the SHAKE algorithm during the entire simulation. The production simulations were extended to 1 000 ps for all structures. RMSF values were analyzed using the ‘Analyze Trajectory’ function in Discovery Studio v19.1.0.18287. Final graphs were obtained with Origin Pro8 v8.0721 (OriginLab Corp., Northampton, MA, USA) (80).

### Circular dichroism spectroscopy

CD spectroscopy experiments were performed on a Chirascan V100 spectrometer using a quartz cuvette with an optical path length of 0.1 cm at 20°C. Spectra were collected between 340 and 200 nm, with a 1s sampling between time points. A blank sample containing only the buffer was treated in the same way and the spectra value was subtracted from the collected data. Oligonucleotide solutions of unliganded duplexes were prepared at a final concentration of 25 μM in buffer containing 20 mM MOPS (pH 6.5), 1 mM Na_2_-EDTA, and 50 mM NaCl. The samples were annealed by heating at 95°C for 10 min and cooled on ice for 30 min. To prepare the liganded DNA:ECHI complexes, 25 μM of duplexes were pre-incubated with 50 μM ECHI at 4°C for 24 h.

### NMR experiments

All NMR experiments were carried out on a BRUKER AVANCE 800 spectrometer equipped with a 5-mm triple resonance cryoprobe and a Z-axis pulsed field gradient at 5°C. For one-dimensional 1H spectra, DNA duplexes samples were prepared at 1 mM in 20 mM NaCl, 20 mM sodium phosphate buffer (pH 7.0) with 10% D_2_O. NOESY experiments, with mixing times of 200 ms, were performed for the same samples but lyophilized and dissolved in 100% D_2_O. All NMR spectra were processed using Bruker Topspin software.

## Results

### Cytosine methylation at non-CpG sites induce polymorphic base pair geometry

**Figure 1.**
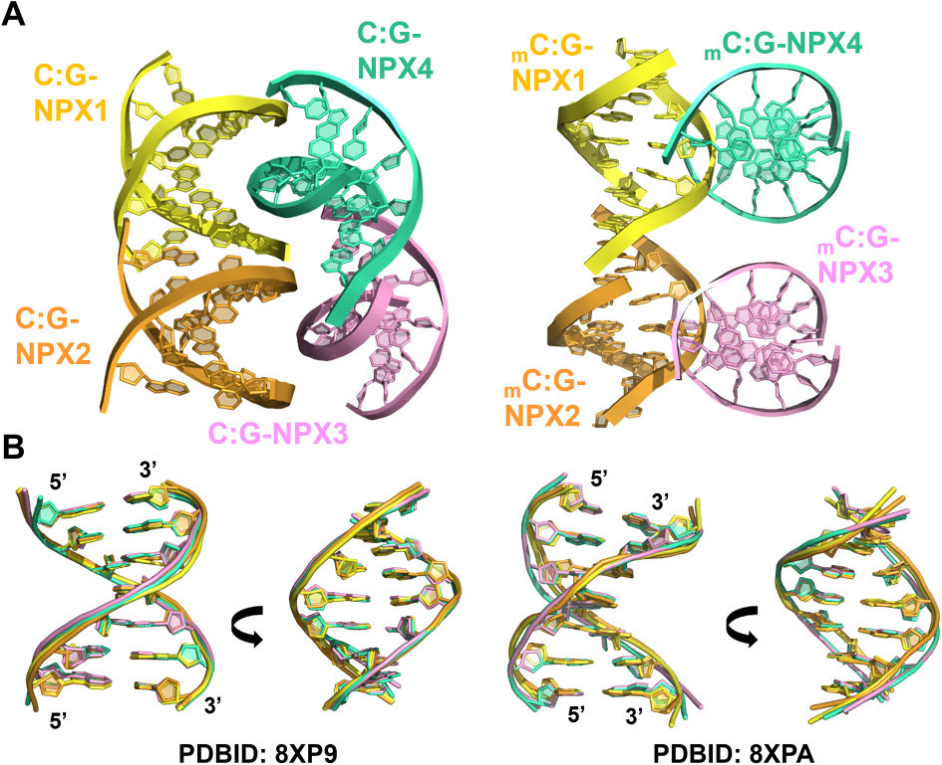
Crystal structures of the unliganded C:G and _m_C:G pairing duplexes. (**A**) Refined structures of unliganded C:G (left) and _m_C:G (Right) pairing duplexes. Each asymmetric unit contains four independent duplexes. DNA duplexes are represented in cartoon form. (**B**) Superimposition of the crystal structures between C:G-NPX1/_m_C:G-NPX1 (yellow), C:G-NPX2/_m_C:G-NPX2 (orange), C:G-NPX3/_m_C:G-NPX3 (pink), and C:G-NPX4/_m_C:G-NPX4 (green) of native (left) and methylated (right) structures. The all-atom root mean square deviations (RMSDs) between C:G-NPX1 and C:G-NPX2, C:G-NPX3 and C:G-NPX4 are 0.2, 0.4, and 0.4, respectively. Meanwhile, the values between _m_C:G-NPX1 and _m_C:G-NPX2, _m_C:G-NPX3 and _m_C:G-NPX4 are 0.4, 0.9, and 0.8, respectively.

The crystal structures of unmethylated d(ACG**C**CGT/ACG**G**CGT) (C:G pairing structure) and methylated d(ACG**_m_C**CGT/ACG**G**CGT) (_m_C:G pairing structure) were solved at resolutions of 2.35 and 2.0 Å, respectively. The data collection statistics are listed in Supplementary Table S1. In these structures, oligonucleotides self-assembled into antiparallel duplexes with four independent duplexes within each asymmetric unit (Figure 1A). The above duplexes were labelled C:G-NPX1 to C:G-NPX4 and _m_C:G-NPX1 to _m_C:G-NPX4 for unmethylated C:G and methylated _m_C:G pairing structures respectively. All duplexes of both the unmethylated and methylated structures were virtually superimposable, with an all-atom root-mean-square deviation (RMSD) of < 1 Å (Figure 1B). The central 5-bp segment of each duplex in both the unmethylated and methylated crystal structures have B-DNA-like duplex conformations. Consistent with the crystal structures, the circular dichroism (CD) spectra of both unmethylated and methylated structures showed negative and positive peaks at 250 and 275 nm, respectively, which are typical for B-DNA (Supplementary Figure S1).

**Figure 2.**
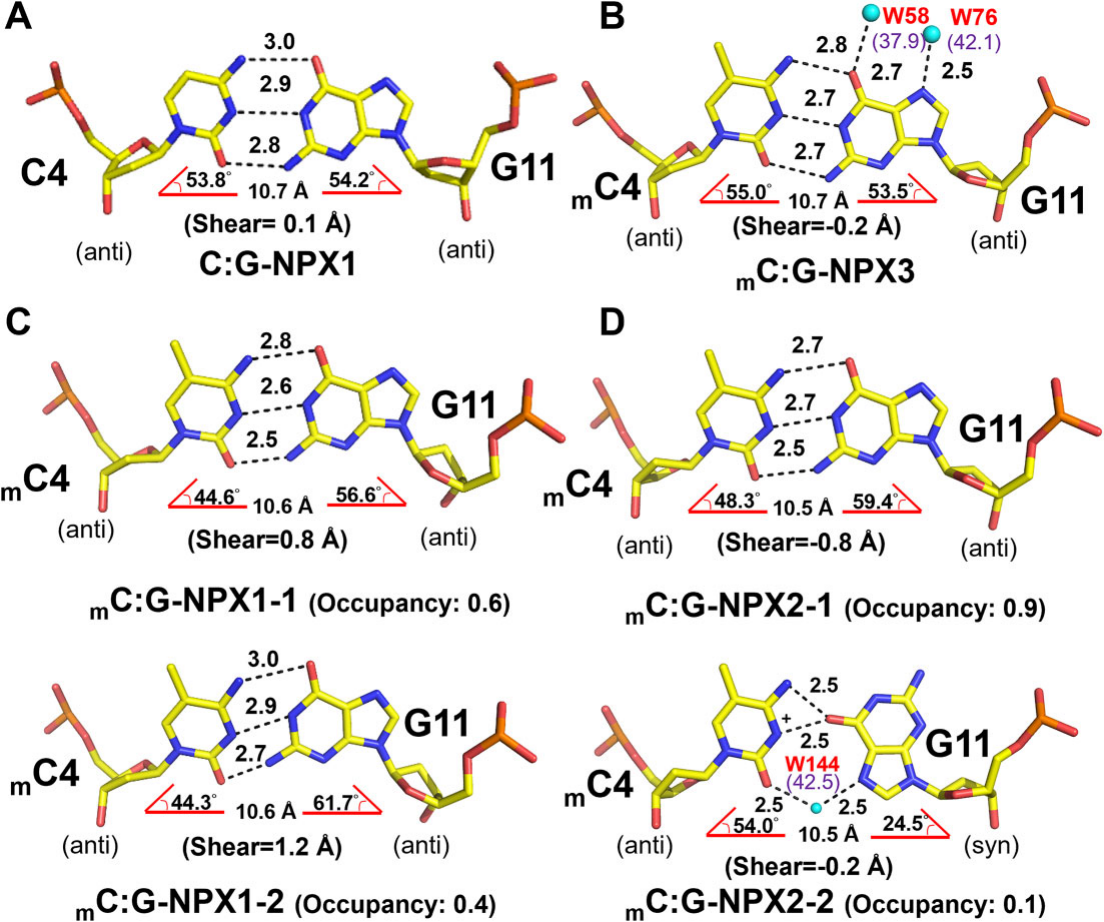
Geometries of the central base pairs in d(ACGCCGT/ACGGCGT)_2_ with or without non-CpG methylated cytosine. Stick representation of the central C4:G11 base pairs of C:G-NPX1 (**A**) and _m_C4:G11 base pair of _m_C:G-NPX3 (**B**), _m_C:G-NPX1 (**C**) and _m_C:G-NPX2 (**D**). Water molecules appear as cyan spheres. Hydrogen bonds are represented by dotted lines, with numbers indicating the distance between the two contributing atoms in angstroms (Å). DNA parameters including C1’–C1’ distance, λ angles, shear and occupancy are indicated at the bottom of the figure. B-factors of each water molecule are indicated with purple letters in brackets.

Despite striking similarities among the unmethylated and methylated structures, we observed several differences between the methylated _m_C4:G11 base pairs and the unmethylated C4:G11 base pairs. To better describe these differences, common parameters including hydrogen bonding patterns, base ‘pivot’ angles (λY for pyrimidines and λR for purines), C1’–C1’ distances, and shearing distances were measured for the _m_C4:G11 and C4:G11 base pairs of each duplex and are listed in Supplementary Table S2 and described in Supplementary Note 1. The measured values suggest that the C4:G11 base pairs in all four unmethylated duplexes adopts a canonical Watson–Crick (WC) conformation (Figure 2A, Supplementary Figures S2A and S11A). On the other hand, six different conformations were observed for _m_C:G base pairs in the methylated duplexes: In _m_C:G-NPX1, the _m_C4: G11 base pair adopts two alternative conformations with occupancy ratios of 0.6 and 0.4, which were designated _m_C:G-NPX1-1 and _m_C:G–NPX1-2, respectively. In _m_C:G-NPX2, _m_C4:G11 base pair also refined to two alternative conformations with occupancy ratios of 0.9 and 0.1, which were thereby designated _m_C:G –NPX2-1 and _m_C:G –NPX2-2, respectively. The central _m_C4:G11 base pairs of _m_C:G–NPX3 and _m_C:G-NPX4 each adopt a WC conformation similar to the unmethylated structure (Figure 2B, Supplementary Figures S2B and S11B).

We further investigated the alternative conformations of the central _m_C4:G11 base pairs in _m_C:G-NPX1 and _m_C:G-NPX2. For _m_C:G-NPX1, both _m_C:G-NPX1-1 and _m_C:G-NPX1-2 adopt an asymmetric Watson–Crick (aWC) conformation (Figure 2C, Supplementary Figures S2C and S11C). Interestingly, for _m_C:G-NPX2, whilst _m_C4:G11 of _m_C:G–NPX2-1 adopts an aWC conformation similar to those observed for _m_C:G–NPX1-1 and _m_C:G–NPX1-2, the same base pair in _m_C:G–NPX2-2 adopts *anti-syn* pairing, in which N4 of _m_C4 forms a hydrogen bond with O6 of G11. Moreover, a water-mediated hydrogen bond is apparent between O2 of _m_C4 and N7 of G11 (Figure 2D, Supplementary Figures S2D and S11D). In addition, a bifurcated hydrogen bond was formed between O6 of G11 and protonated N3 of _m_C4 to further stabilize this *anti*-*syn*pairing. We have designated the unique geometry observed in _m_C:G–NPX2-2 as (w)cWH to represent a water-mediated *cis* Watson–Crick/Hoogsteen base pair geometry (81,82) (Supplementary Note 1). In summary, the base pair geometries of the central _m_C4:G11 in the methylated duplexes fall into three types: WC (_m_C:G–NPX3 and _m_C:G –NPX4), aWC (_m_C:G–NPX1-1, _m_C:G–NPX1-2, and _m_C:G–NPX2-1), and (w)cWH (_m_C:G–NPX2-2).

### Polymorphic base pair geometry resulted from non-CpG methylation rather than solely from WC base pair instability

The methylation-site polymorphisms suggested that cytosine methylation may enhance the dynamics of modified base pairs. In light of this, we performed MD simulations on the C:G–NPX1, _m_C:G–NPX3, _m_C:G–NPX2-1 and _m_C:G–NPX2-2 duplexes to investigate the impact of cytosine methylation on base flexibility, and calculated the toot-mean-square fluctuation (RMSF) values in each structure to represent the flexibility of individual bases. The results showed that the largest fluctuation occurred at the central base of the methylated structure compared to the unmethylated structure (Supplementary Figure S3A). This indicates that cytosine methylation enhanced the base pair dynamics of methylated sites compared to the unmethylated structure, consistent with the high degree of polymorphism in methylated base pairs observed in the crystal structure.

To further test whether base pair dynamics are primarily responsible for the polymorphic base pair geometry that promote (w)cWH formation, we replaced the guanine of the center _m_C:G pair with inosine. Since the only difference between guanine and inosine is the absence of the N2 amino group in inosine, this substitution is ideal for reflecting the base pair instability associated with the loss of a single H-bond (83). The unliganded C:I pairing structure was determined at a resolution of 2.5 Å. Each asymmetric unit contains four independent duplexes, labelled C:I-NPX1 to C:I-NPX4. The central C4:I11 base pairs in all four duplexes adopt a single *anti*–*anti*pair in a non-canonical WC geometry (Supplementary Note 1 and Supplementary Figures S3B and S11E). This could be attributed to the instability of WC pairing due to the loss of H-bonds. However, we did not observe any alternative conformations in C:I-NPXs. To investigate the impact of inosine substitution on base flexibility, we performed MD simulation on the C:I-NPX1 structure. The choice of C:I-NPX1 as the representative structure was because its central C4:I11 base pair has parameter values that are close to the average values of the four duplexes. Our results showed that higher fluctuation was observed at the central bases of the C:I-NPX1 compared to the native unmethylated structure (Supplementary Figure S3A). This indicates that substitution of guanine by inosine enhanced base pair dynamics. Overall, the above results indicate that the instability of WC pairing could lead to higher base pair dynamics, while cytosine non-CpG methylation is mainly responsible for the observed polymorphic base pair geometry and (w)cWH formation.

### Structural basis of methylation-induced polymorphism at the _m_C4:G11 position

**Figure 3.**
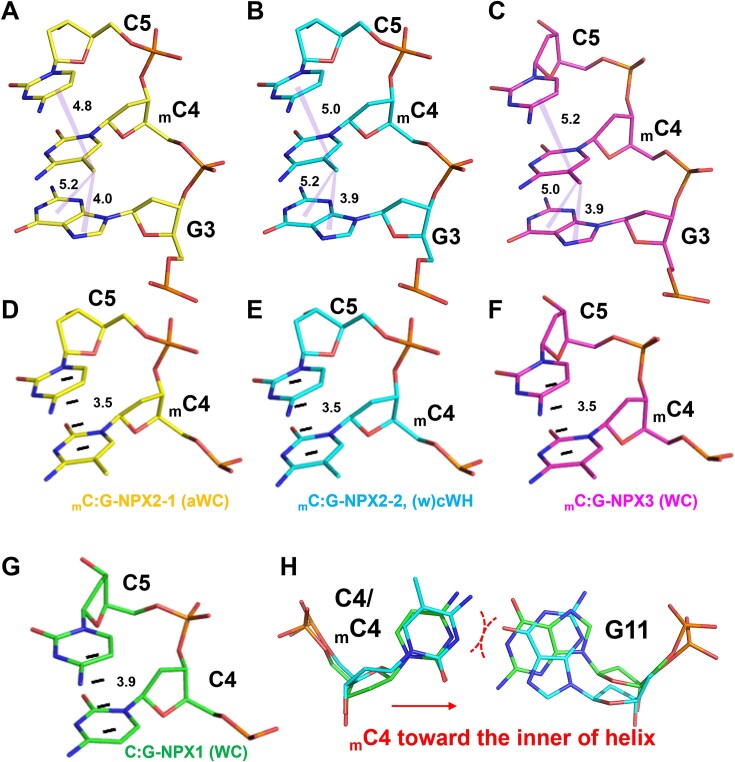
Effect of stacking interactions involving 5_m_C on the geometry of DNA base pairs. (A–C) Stacking of methyl groups with G3 and C5 bases in _m_C:G-NPX2-1 (**A**), _m_C:G NPX2-2 (**B**) and _m_C:G-NPX3 (**C**) structures. (D–G) π–π stacking between _m_C4/C4 and C5s in C:G-NPX1 (**D**), _m_C:G-NPX3 (**E**), _m_C:G-NPX2-1 (**F**) and _m_C:G-NPX2-2 (**G**) are indicated by the black dashed lines. (**H**) Changes of mC4 position sterically destabilize _m_C:G WC bps (red dashes) and favor the (w)cWH base pair formation.

As shown above, cytosine methylation at the non-CpG step may result in geometric polymorphism of methylated base pairs. The structural basis of this polymorphism may be deduced through a detailed analysis of the interaction differences among the unmethylated and methylated conformations. Because the geometry of the C4:G11 base pair in all four unmethylated duplexes were similar, C:G-NPX1 was selected as a representative of the unmethylated conformation. Likewise, _m_C:G–NPX3, _m_C:G–NPX2-1 and _m_C:G–NPX2-2 were chosen as representatives of duplexes containing WC, aWC, or (w)cWH base pairs, respectively. Figure 3A–C shows the methyl–π stacking interactions between the methyl groups of _m_C and adjacent nucleotides in the different methylated structures; interactions which were not available to the unmethylated DNA. The _m_C4 and C5 bases were better aligned when compared to their unmethylated counterparts. The π–π distances between the _m_C4 and C5 bases in the methylated structures (3.5 Å) were also shorter than that in the unmethylated structure (3.9 Å) as shown in Figure 3D–G. These results suggest stronger stacking interactions in the methylated structures compared to the unmethylated structure. On the other hand, the distances between the fifth carbon of C4 and the sugar ring of the G3 base in the methylated structures were longer than those in the unmethylated counterparts (Supplementary Figure S4A–D). This difference is likely attributed to steric hindrance between the methyl group and the phosphate backbone, causing the methylated cytosine to move towards the interior of the helix. The repositioning of _m_C4 sterically hinders WC geometry, making the methylated WC more flexible and facilitating the formation of (w)cWH geometry (Figure 3H).

The different methylated structures were further stabilized through distinct networks of metal ions and water molecules around their methylated base pairs (Supplementary Figure S4E-F). In _m_C:G-NPX3, G3 and _m_C4 are paired indirectly to each other through the W75- and W127-mediated interactions (Supplementary Figure S4E). A Mn^2+^ coordinates with two symmetry-related N7 atoms of G10 and four water molecules W88, W57, W128, and W58 in a perfect octahedral geometry. This cluster of Mn^2+^ ion and water molecules stabilize the methylated base pairs of _m_C:G-NPX3 in a symmetrical WC conformation. In contrast, only one water molecule (W141) interacts with N7 of G3 and N4 of _m_C4 in both _m_C:G-NPX2-1 and _m_C:G-NPX2-2 (Supplementary Figure S4F). The smaller number of water-mediated interactions may provide more flexibility to the methylated base pair in _m_C:G-NPX2 compared to that in _m_C:G-NPX3, which may explain why the methylated base pair in _m_C:G-NPX2 could adopt two alternative conformations. In addition, the lack of water-mediated interactions stabilizing G10 in _m_C:G-NPX2-1 may partially explain why it assumes an aWC conformation. In addition, we further observed that the stacking interaction between G11 and G10 seems to play a role in determining the conformation of G11. In the _m_C:G-NPX2-1 structure, G11 is in the *anti*-form and aligned with G10 in the same direction, whereas in the _m_C:G-NPX2-2 structure, G11 is in the *syn*-form and is aligned with G10 in the opposite direction, which may result in different stacking energies (Supplementary Figure S5). This could account for the difference in occupancy of the aWC and (w)cWH geometries in the _m_C:G-NPX2 duplex.

### (w)cWH base pair formation can be stabilized by echinomycin intercalation

**Figure 4.**
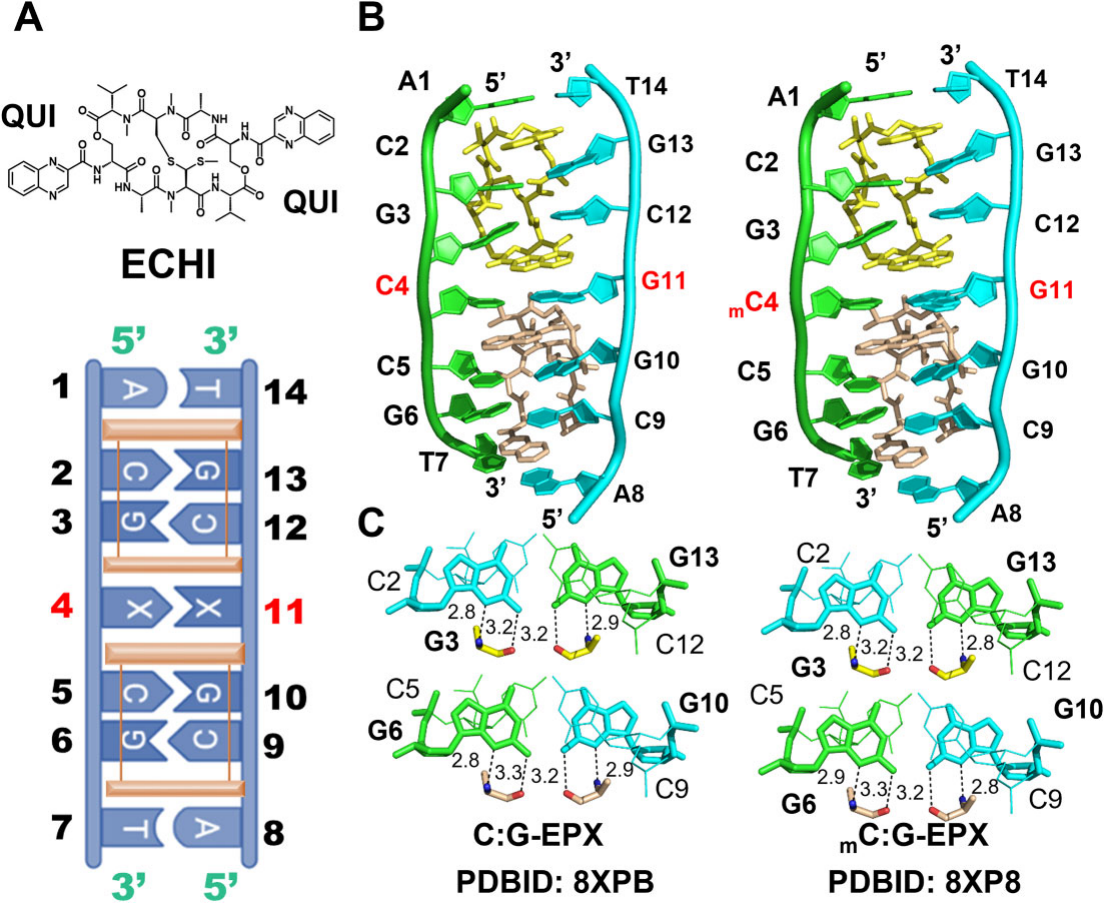
Crystal structures of DNA:ECHI complex with or without methylated cytosine. (**A**) (upper) Chemical structure of ECHI. (lower) Schematic representation of the DNA:ECHI complex. Central X4:X11 (highlighted in bold red) represent different central base pairs used in this study. ECHI intercalated between the C2pG3/C5pG6 steps is represented in orange. (**B**) Overall structures of C:G-EPX (left) and _m_C:G-EPX (Right) complex. ECHI1 and ECHI2 are colored in yellow and orange, respectively. (**C**) Hydrogen bonds between ECHI and DNA at the CpG step of C:G-EPX (left) and _m_C:G-EPX (Right) complexes. Dotted lines represent direct interactions between atoms, and numbers indicate the distances.

Since structural analysis indicated that stacking interaction between the guanine of the methylated _m_C:G base pair and the flanking bases plays an important role in stabilizing the guanine, we tried to further stabilize the methylated _m_C:G base pair using the drug echinomycin (ECHI). A single ECHI molecule intercalates into both sides of a 5′-CpG DNA step and forms strong stacking interactions with bases flanking the intercalation site (84) (Figure 4A). The crystal structures of C:G and _m_C:G duplexes in complex with ECHI were designated C:G-EPX and _m_C:G-EPX, and solved at 2.0 and 1.64 Å resolution, respectively (Supplementary Table S1). Overall, the two structures are closely similar, with an all-atom RMSD of 0.18 Å, and their conformations resemble previously proposed DNA:ECHI complex structures (74) (Figure 4B). The CD spectra of C:G-EPX and _m_C:G-EPX structures revealed that these structures are nearly identical in solution (Supplementary Figure S6), on accord with the results of the crystal structure analysis. Each asymmetric unit contains a DNA duplex bound by two ECHI molecules, labeled ECHI 1 and ECHI 2. The two planar quinoxaline rings of each ECHI molecule flank the CpG steps in the minor groove (Figure 4B). The details of ECHI binding to C:G and _m_C:G duplexes are consistent with previously reported echinomycin-DNA interactions (Figure 4C and Supplementary Note 2).

**Figure 5.**
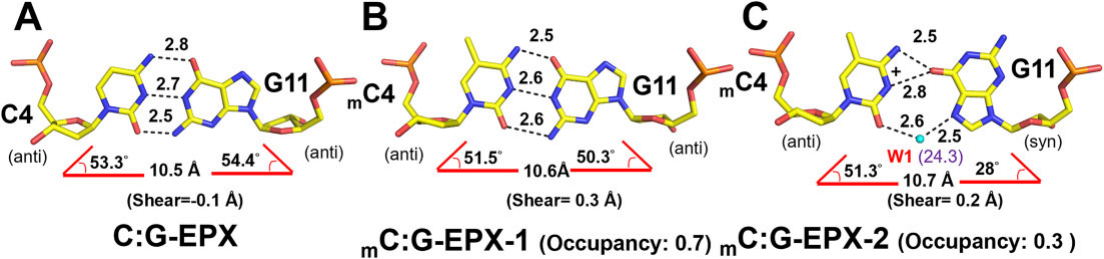
Central base pair geometry in C:G-EPX and _m_C:G-EPX complexes. (A–C) Stick representation of the central base pairs of C:G-EPX (**A**), _m_C:G-EPX-1 (**B**) and _m_C:G-EPX-2 (**C**). Water molecules appear as cyan spheres. Hydrogen bonds are represented by dotted lines, with numbers indicating the distance between two contributing atoms in angstroms (Å). DNA parameters including C1’–C1’ distance, λ angles, shear and occupancy are indicated at the bottom of the figure. B-factors of each water molecule are indicated with purple letters in brackets.

The geometries of the _m_C4:G11 and C4:G11 base pairs in each complex are fully described in Supplementary Note 2, with the parameters listed in Supplementary Table S2. The C4:G11 base pair in the C:G-EPX structure adopts a symmetrical WC conformation similar to that of the C:G-NPX1 structure (Figure 5A, Supplementary Figures S7A and S12A). On the other hand, the _m_C4:G11 base pair in the _m_C:G-EPX structure exhibits two alternative conformations with occupancies of 0.7 and 0.3, designated _m_C:G-EPX-1 and _m_C:G-EPX-2, respectively. The _m_C4:G11 base pair in _m_C:G-EPX-1 adopts a symmetrical WC conformation similar to the unmethylated structure (Figure 5B, Supplementary Figures S7B and S12B). The central base pair of the _m_C:G-EPX-2 structure adopts a geometry similar to (w)cWH (Figure 5C, Supplementary Figures S7C and S12B). The *syn*-G11 in _m_C:G-EPX-2 was found to stack better with the QUI ring of ECHI2 compared to *anti*-G11 in C:G-EPX and _m_C:G-EPX-1 (Supplementary Figure S8A–C). In addition, water molecules in the vicinity of _m_C4:G11 are organized differently in C:G-EPX, _m_C:G-EPX-1 and _m_C:G-EPX-2 (Supplementary Figure S8D–F). A single conserved water molecule is present in both C:G-EPX and _m_C:G-EPX complexes (W8 in C:G-EPX and W6’ in both _m_C:G-EPX-1 and _m_C:G-EPX-2). This water molecule was found to mediate the indirect interactions between N4 of the ECHI1 quinoline ring and O6 of G11 in both the C:G-EPX and _m_C:G-EPX complexes. In the latter complexes, W6’ also bridges N4 of the ECHI1 quinoline and N4 of _m_C4, further stabilizing the position of the _m_C4 base. Three additional water molecules, W1, W6 and W41, were observed only in the methylated complexes. W41 is the only one common to both _m_C:G-EPX-1 and _m_C:G-EPX-2, and mediates the interaction between N4 of the ECHI2 quinoline and O6 of G11 (Supplementary Figure S8B, C). W1 and W6 are unique to _m_C:G-EPX-2. W6 interacts with W41 and N1 of G11, helping to stabilize G11 in the *syn*-conformation. W1 interacts with O2 in _m_C4 and N7 in G11, further helping to stabilize the unusual geometry of the (w)cWH base pair in _m_C:G-EPX-2. A hydrogen bond was also found between N2 and OP2 of G11, which stabilizes the *syn-*conformation of G11 in _m_C:G-EPX-2 (Supplementary Figure S8C). Collectively, these extra interactions may play a significant role in stabilizing the _m_C4:G11 base pair in _m_C:G-EPX-1 and _m_C:G-EPX-2, while the unique hydrogen bonds observed in _m_C:G-EPX-2 may account for the increased occupancy of the (w)cWH geometry observed in ECHI-bound DNA.

### Alternative conformations are observed for non-CpG methylated duplexes in solution

**Figure 6.**
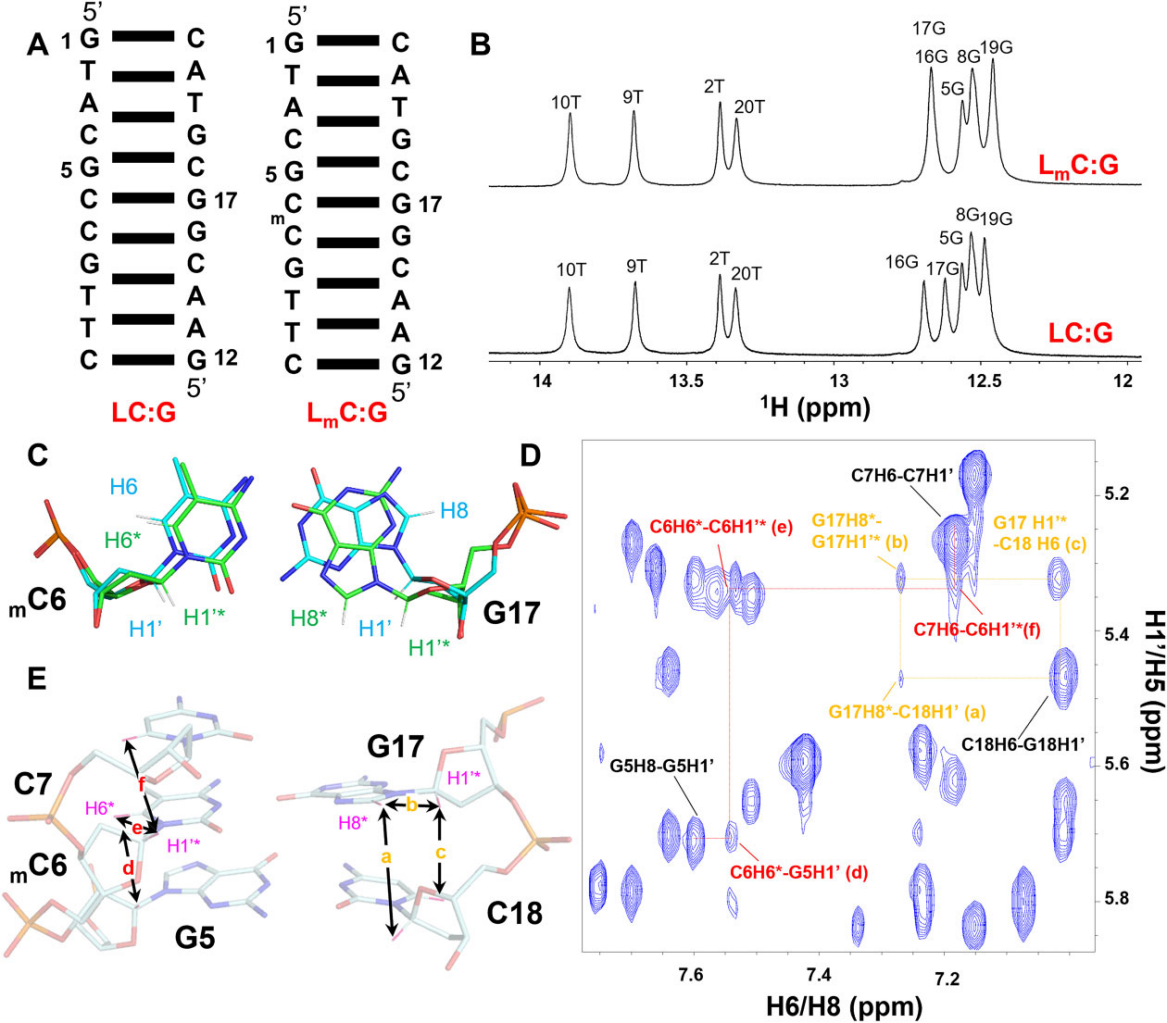
non-CpG methylation-induced alternative conformations in solution. (**A**) LC:G and L_m_C:G DNA duplexes used in this study. (**B**) 1D imino 1 H spectra of LC:G (lower) and L_m_C:G (upper). Spectra were measured in 20 mM NaCl, 20 mM PBS buffer (pH = 7) at 5°C, and 1 mM DNA duplex concentrations. (**C**) Schematic illustration of the H8, H6, and H1’ protons in major (blue) and alternative (w)cWH (green) conformations of the methylated base-pair. (**D**) 2D 1H, 1H NOESY spectra of L_m_C:G. Six crucial NOEs characteristic of an alternative (w)cWH conformation of _m_C6:G17 base-pair, as indicated in (**E**) were marked with lowercase letters a–f in brackets.

The non-CpG methylation-induced base pair dynamics and (w)cWH formation were monitored by NMR experiments. To avoid the fraying effect at the terminal bases that could impair the DNA double helix conformation during NMR experiments, we incorporated two additional base pairs flanking the _m_C:G/C:G pairing duplexes to enhance their stability (Figure 6A). The duplexes, d(GAACGCCGTAC/GTACGGCGTTC)_2_ and d(GAACG_m_CCGTAC/GTACGGCGTTC)_2_, were designated LC:G and L_m_C:G, respectively. Figure 6B. shows the 1D 1H NMR spectra of L_m_C:G and LC:G structures. From the spectra, it is clear that the unmethylated LC:G structure most likely form a duplex, confirmed by the observation of six sharp signals in the region between 12 and 13 ppm, as well as four signals within the 13 to 14 ppm range. These signals are characteristic of the H1 proton of a guanine residue and the H3 proton of thymine involved in WC base pairs, respectively. The spectrum of the methylated L_m_C:G structure exhibited a similar signal pattern in the imino region, except for the signals observed between 12.65 and 12.75 ppm. The signals of the G16 and G17 imino protons, which occur at 12.71 and 12.65 ppm in the LC:G spectrum, shifted and overlapped into one signal at 12.73 ppm in the L_m_C:G spectrum (Figure 6B). This observation suggests that the overall folds of both duplexes were similar with the exception of G16 and G17, which were affected by the presence of the methyl group in _m_C.

To further explore the conformational differences induced by non-CpG methylation, two-dimensional nuclear Overhauser effect spectroscopy (NOESY) experiments were performed. Supplementary Figures S9A and B shows the aromatic to H1’/H5 region of the NOESY in D_2_O for the LC:G and L_m_C:G structures, respectively, which were assigned using well-established strategies (85). A comparison of the NOE patterns between LC:G and L_m_C:G within this region is shown in Supplementary Figure S9C. Four additional NOEs (peaks a-b and d-e), none of which were part of sequential walks, were observed in the L_m_C:G spectrum, indicating that an additional conformation might be present in the methylated duplex. We inferred that these additional signals may belong to the alternative (w)cWH conformation of the methylated base-pair. This inference was confirmed by the six NOEs, correlated to the H6 and H1’ protons in cytosine, as well as H8 and H1’ protons in guanine of the alternative (w)cWH conformation of methylated base-pair (Figure 6C). These include the four unique NOEs observed only in L_m_C:G spectrum: between the G17H8* proton and the C18H1’ proton (cross-peak a), the G17H8* proton and the G17H1’* proton (cross-peak b, which was overlapped with the NOE between A3H2 and C4H1’), the C6H6* proton and the G5H1’ proton (cross-peak d) and between the C6H6* proton and the C6H1’* proton (cross-peak e). As well as the NOE exhibited from C7H6 proton to the C6H1’* proton (cross-peak f), and which exhibited from G17H1’* proton to C18H6 proton (cross-peak c, overlapped with the NOE exhibited from C4H8 to C4H1’) (Figure 6D-E). In the alternative (w)cWH conformation of the methylated base-pair, it was observed that G17 adopted a *syn*-conformation. This was supported by the G17H8*−C18H1’ connectivity (peak a), which is typical for *syn-anti* step of a sequential walk (Figure 6E). The occurrence of an alternative conformation suggested non-CpG methylation enhanced dynamics of the methylated base-pair is consistent with our crystallography results. In total, the NMR data provide a hint of the existence of an *anti*→*syn* transition of guanosine in the non-CpG methylated _m_C:G base pair in solution, which may indicate (w)cWH formation.

## Discussion

Several previous studies have shown that cytosine methylation affects base flexibility, which in turn impacts DNA local structure (86–88). To investigate the structural impact of non-CpG methylation on the structure of duplex DNA, we have determined the crystal structures of DNA sequences associated with neurodevelopmental disorder with or without a non-CpG methylation site. The crystal structure of the methylated _m_C:G duplex has revealed that cytosine methylation at non-CpG site results in geometric polymorphism of the methylated _m_C:G base pair, which leads to the formation of a unique (w)cWH geometry with a *syn*-guanosine in a population of around 10%. This phenomenon was also confirmed by NMR spectroscopy in aqueous solution. Molecular dynamics (MD) simulations of the unliganded structures further demonstrate that non-CpG methylation may enhance the dynamics of methylated base pairs. These findings imply that in a non-CpG context, the methylated cytosine is highly dynamic which in turn may allow the *anti*→*syn* transition of its complementary guanosines, resulting in the formation of the (w)cWH geometry.

The source of the highly dynamic character of the methylated cytosine may originate from several causes. The identity of the 3′-neighboring base next to the methylation site may play an important role because a bulkier base (e.g. G or A) may constrain the dynamic freedom of the methylated cytosine. The transition from WC to (w)cWH also requires a large space, which may be defined by the intra-strand phosphate-to-phosphate (P-P) distances of adjacent nucleotides. B-DNA has the largest P-P distance of all forms of DNA, and may be a prerequisite for the conformational transition to occur (66). On the other hand, base dynamics of the methylated cytosine may not be sufficient to form the (w)cWH geometry. Using the unmethylated sequence, we introduced additional base pair instability by substituting the guanosine with an inosine (83), which results in the loss of an H-bond, leading to only a single base pair geometry in the structure, even though the dynamics of the C:I base pair had been enhanced compared to the original sequence. This suggests that either the base dynamics were not enhanced to a sufficient degree to allow the (w)cWH geometry to occur, or that base dynamics alone may not be sufficient to cause the (w)cWH geometry to form.

Further clues lie in the atomic interactions between the bases surrounding the methylation site. Our structural analyses showed that in the non-CpG step, the stacking interactions between _m_C:G base pair and its adjacent nucleotides are crucial for stabilizing the (w)cWH configuration. For example, the bulky base at the G10 position of our DNA sequence constrains G11 to be in the *syn*-conformation. This is corroborated by the observation that the echinomycin-bound structures, which have enhanced stacking interactions caused by ligand intercalation, have higher (w)cWH base-pair occupancies compared to the unliganded structures. Taken together, stabilization of the (w)cWH geometry appears to employ all the hallmark mechanisms found in the stabilization of a typical Hoogsteen base pair.

Based on our findings, an analysis of all available structures of unliganded DNA duplexes containing methylated cytosines may provide clues on why the (w)cWH geometry has never been previously reported (Supplementary Table S3) (36,38,39,73,89–92). Among all DNA sequences studied, that used in this study is the only one that fulfilled all of the following criteria: (a) having the dynamic freedom of the methylated cytosine; (b) forms a B-form DNA; (c) the Hoogsteen base pair has favorable stacking interactions between the methylated base pair and adjacent bases. Accordingly, we propose that the (w)cWH geometry may be specific to base steps CpC and CpT, which fulfils all of the above criteria (Supplementary Figure S10). Our crystal structures provide the first insight of the alternative (w)cWH geometry arising from non-CpG methylation.

X-ray crystallography is a powerful tool for identifying alternative conformations and pinpointing the atomic positions of water molecules in biological macromolecules (93,94). Miao and Cao analysed protein structures containing alternative side-chain conformations and concluded that alternative conformation could be observed in crystal structures when the resolution is better than 2.0 Å (95), further rationalizing the discovery of alternative conformations in our high-resolution data. In the unliganded (w)cWH base pair, the electron density associated with one water molecule at 0.12 occupancy may be insufficient to confidently determine its precise position. However, with the increased occupancy of 0.32 upon stabilization of (w)cWH by ECHI, a highly similar geometry as well as water location accompanied by a clearer map was observed. This strengthens the credibility of the water molecule location within the (w)cWH in both the unliganded and liganded structures.

**Figure 7.**
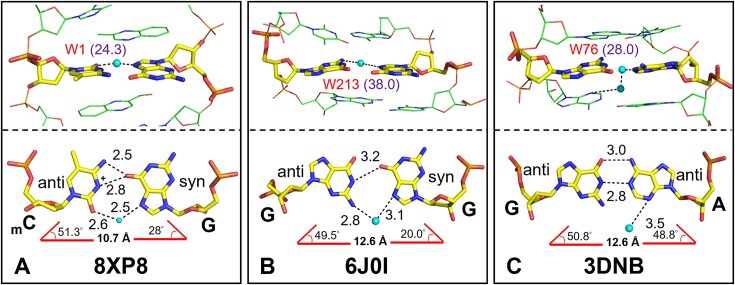
The role of water molecules in stabilizing non-canonical base pairs. Water-mediated interactions stabilize unusual base pair geometries by avoiding steric clashes between bases. Non-canonical base pairs are shown in yellow sticks. Water molecules appear as a cyan spheres. Hydrogen bonds are represented by dotted lines, with numbers indicating the distance between two contributing atoms in angstroms (Å). The C1’–C1’ distance and λ angles are indicated at the bottom of the figure. The respective PDB-ID for each structure is given in bold black letters. *B*-factors of each water molecule are indicated with purple letters in brackets.

The importance of water cannot be overstated. Previous studies have shown that cytosine methylation affected the hydration pattern around the methylated sites, which may be important for protein recognition (96,97). In this work, although the *syn-*guanosine is engaged in Hoogsteen-edge pairing with methylated cytosine to reduce steric clashes between the bases, the longer C1’–C1’ distance results in weaker hydrogen bonds compared to canonical HG base pairs. These weaker hydrogen bonds are compensated by water-mediated interactions (Figure 7A). Other studies have shown that two mismatches with steric clashes, G:G and G:A, can generate unusual base-pair geometries through water-mediated pairing, with the G:G mismatch adopting a water stabilized *anti–syn* geometry (Figure 7B) (54), and the G:A mismatch adopting an unusual geometry with a large propeller twist mediated by two water molecules to avoid steric clashes between mismatched bases (Figure 7C) (98). These results imply that unusual base pairings that are stabilized through water-mediated interactions may be more prevalent than one might expect.

The ability of non-CpG methylated DNA to adopt the (w)cWH geometry may have important functional consequences in epigenetic regulation. Several cancers have been associated with methylation status changes at non-CpG sites of the genome (10,32,99). Although spontaneous deamination is usually invoked as the major mutagenic mechanism in these cases, our findings allude to the intriguing possibility of an alternative mutagenic mechanism: methylation at the non-CpG sites may promote formation of (w)cWH base pairs, which are less stable than WC base pairs and have been shown to increase the susceptibility of double-stranded DNA to damage (100,101). The (w)cWH base pairs may also affect gene regulation because Hoogsteen base pairs are known to play important roles in DNA-protein recognition (59,60,63,65).

In summary, we have identified a novel (w)cWH geometry that may form part of the conformational ensemble specific to non-CpG methylated DNA. We propose that this (w)cWH geometry may provide an alternative step in the mechanism for methylation-mediated genetic instability and may have important functional consequences in epigenetic regulation. This study thus provides the first structural insights into the formation of alternative base pair geometries in non-CpG methylation.

## Supplementary Material

gkae594_Supplemental_File

## Data Availability

All data are available in the main text or the supplementary materials. Structures have been deposited in the PDB (https://www.rcsb.org/) under accession numbers 8XP9 (unliganded C:G pairing structure), 8XPA (unliganded _m_C:G pairing structure), 8WNB (unliganded C:I pairing structure), 8XPB (C:G-ECHI complex) and 8XP8 (_m_C:G-ECHI complex).
