# Supplementary material for: Structural basis of water-mediated cis Watson–Crick/Hoogsteen base-pair formation in non-CpG methylation
Source: Nucleic Acids Res. 2024 Jul 11;52(14):8566–79. doi: 10.1093/nar/gkae594 (PMC11317146; doi:10.1093/nar/gkae594)
Supplement: gkae594_Supplemental_File [file gkae594_supplemental_file.pdf]

Supplementary Data

Structural basis of water-mediated cis Watson-Crick/Hoogsteen base-pair formation in non-CpG methylation

Shan-Meng Lin[1], Hsiang-Ti Huang[1], Pei-Ju Fang[2], Chi-Fon Chang[3], Roshan Satange[1], Chung-ke Chang[4], Shan-Ho Chou[5], Stephen Neidle[6*], Ming-Hon Hou[1,7,8,9,10*]

[1]Institute of Genomics and Bioinformatics, National Chung Hsing University, Taichung, 402, Taiwan
[2]Institute of Biomedical Sciences, Academia Sinica, Taipei 115, Taiwan
[3]Genomics Research Center, Academia Sinica, Taipei 115, Taiwan
[4]Taiwan Biobank, Institute of Biomedical Sciences, Academia Sinica, Taipei 115, Taiwan
[5]Institute of Biochemistry, National Chung Hsing University, Taichung 402, Taiwan
[6]School of Pharmacy, University College London, London, WC1N 1AX, UK
[7]Ph.D. Program in Medical Biotechnology, National Chung Hsing University, Taichung402, Taiwan
[8]Graduate Institute of Biotechnology, National Chung Hsing University, Taichung402, Taiwan
[9]Department of Life Sciences, National Chung Hsing University, Taichung 402, Taiwan
[10]Biotechnology Center, National Chung Hsing University, Taichung 402, Taiwan

[*]To whom correspondence should be addressed. Tel: +886 4 2284 0338 (Ext 7011); Email: s.neidle@ucl.ac.uk
[*]Correspondence may also be addressed to Ming-Hon Hou. Tel: +886 4 2284 0338 (Ext 7011); Fax: +886 4 2285 9329; Email: mhho@nchu.edu.tw

Supplementary Note 1. Structure details of central base pairs in unliganded DNA duplexes

To better describe the geometric differences between methylated mC4:G11 base pairs and unmethylated C4:G11 base pairs, we applied common parameters of base pairs to define their geometries, as explained below: In a canonical C:G WC base pair, the λY and λR angles of cytosine and guanine are 54.2° and 54°, respectively (51); the C1'–C1' distance is 10.8 Å (51); and the average shear distance is 0 Å (51). These values represent a symmetric base-pair geometry of canonical WC, which is essential for maintaining base pair stability. Accordingly, larger values of shear distance and λ angle differences (the difference between λY and λR) can serve as indicators of greater instability of the base pair and a propensity towards non-WC geometry. Consequently, such parameters are commonly found in other non-WC geometries, like mismatches (52). Therefore, to further investigate the non-WC base pair geometry caused by non-CpG methylation, in this study, we define the base pair geometry with λ angle differences ≤ 4 and shear distance ≤ 0.3 as WC, and the base pair geometry with λ angle differences ≥ 10 and shear distance ≥ 0.8 as asymmetric Watson-Crick conformation (aWC) to represent an unusual and relatively asymmetric geometry compared to canonical WC base pairs.

In the unmethylated C:G pairing structure, the central C4:G11 base pairs in all four duplexes adopt a single anti–anti conformation with hydrogen bonding pattern similar to those in canonical WC conformation, namely, the N4, N3, and O2 atoms of cytosine form three hydrogen bonds with the O6, N1, and N2 atoms of guanine respectively. The λY and λR angles of C4s and G11s are ca. 53° and 53–56° respectively, in all four duplexes of the unmethylated structure. The interstrand C1'–C1' widths of these C4:G11 base pairs in all four unmethylated duplexes are ca. 10.5-10.7 Å. These C4:G11 base pairs have a shear distance below 0.3 Å (Supplementary Table S2). These parameter values suggest that the central C4:G11 base pair in all four duplexes of the unmethylated structure adopts a canonical WC base pair conformation. For the methylated $_{m}$C:G pairing structure, in $_{m}$C:G-NPX1, the $_{m}$C4:G11 base pair adopts two alternative conformations with occupancy ratios of 0.6 and 0.4, which are designated $_{m}$C:G-NPX1-1 and $_{m}$C:G–NPX1-2, respectively. Both conformations adopt an *anti–anti* hydrogen bonding pattern similar to that in canonical WC. However, the λY and λR angles for $_{m}$C4 and G11 in the two conformations are ca. 44° and 57-62°, respectively. This suggests that the positions of $_{m}$C4 and G11 in the methylated $_{m}$C4:G11 base pairs of $_{m}$C:G-NPX1-1 and $_{m}$C:G-NPX1-2 are relatively asymmetric compared to the position in the unmethylated C4:G11 base pairs of C:G-NPX1. Additionally, the $_{m}$C4:G11 base pairs have shear distances of 0.8 and 1.2 Å in $_{m}$C:G-NPX1-1 and $_{m}$C:G-NPX1-2 respectively. These larger shear distances show that the guanine base in the methylated base pair is positioned closer to the major groove than that in C:G-NPX1. Thus, the pivot angle and shear distance values suggest that the

methylated base pairs in $_{m}$C:G-NPX1 adopt a relatively asymmetric conformation compared to the canonical WC base pair (Supplementary Table S2). The unusual and relatively asymmetric conformation of the central $_{m}$C4:G11 base pair in $_{m}$C:G-NPX1-1 and $_{m}$C:G-NPX1-2 compared to the canonical WC base pair suggests that the above parameter values are characteristic of the aWC conformation.

On the other hand, the central base pair of $_{m}$C:G –NPX2-1 adopts an *anti–anti* hydrogen bonding pattern similar to canonical WC. The λY and λR values for $_{m}$C4 and G11 of $_{m}$C:G–NPX2-1 are 48° and 59°, respectively. The $_{m}$C4:G11 base pair has a shear distance of -0.8 Å, and the C1'–C1' interstrand distance for $_{m}$C4:G11 is 10.6 Å. All of the above parameter values suggest that the central $_{m}$C4:G11 base pair of $_{m}$C:G –NPX2-1 adopts an aWC conformation similar to those of $_{m}$C:G–NPX1-1 and $_{m}$C:G–NPX1-2. While the central base pair of $_{m}$C:G–NPX2-2 adopts an *anti-syn* pairing, and N4 of $_{m}$C4 form a hydrogen bond with O6 of G11. Moreover, a water-mediated hydrogen bond is formed between O2 of $_{m}$C4 and N7 of G11. The λY and λR angles for $_{m}$C4 and G11 in $_{m}$C:G –NPX2-2 were 51.6° and 24.7°, respectively. The shear distance was -0.2 Å and the C1'–C1' width of the base pair was 10.7 Å (Supplementary Table S2). These parameters suggest that the central $_{m}$C:G pair in $_{m}$C:G–NPX2-2 exhibited a noncanonical base-pair geometry due to the formation of hydrogen bonds between the N4 atom of WC-edge cytosine and the O6 atom of Hoogsteen (HG)-edge guanine, and the cis orientation of the glycosidic bonds as well as the presence of a water molecule that stabilized this unique base pair. Furthermore, a previous study has shown that the pK_a of cytosine N3 within a duplex DNA is around 7.2 (68), suggesting that the N3 of $_{m}$C4 may be protonated in our study. In this case, a bifurcated hydrogen bond could be formed between O6 of G11 and the protonated N3 of $_{m}$C4 to further stabilize this anti-syn pairing. Based on the base pair arrangement, we have designated this unique geometry type as water-mediated cis Watson-Crick/Hoogsteen geometry, abbreviated as (w)cWH (81-82). On the other hand, the central base pairs of $_{m}$C:G–NPX3 and $_{m}$C:G–NPX4 adopt a single *anti–anti* conformation with hydrogen-bonding patterns similar to those in canonical WC. The λY and λR angles for $_{m}$C4 and G11 in $_{m}$C:G–NPX3 and $_{m}$C:G –NPX4 were ca. 53-58° and 54°, respectively. $_{m}$C:G–NPX3 and $_{m}$C:G–NPX4 have a C1'–C1' width distance of 10.7 Å and a shear distance of 0.2 Å (Supplementary Table S2). These parameters suggest that the central $_{m}$C4:G11 base pairs of $_{m}$C:G–NPX3 and $_{m}$C:G-NPX4 adopt a WC conformation similar to the unmethylated structure.

For the unliganded C:I pairing structure, the central C4:I11 base pairs in all four duplexes adopt a single anti–anti pair, forming two hydrogen bonds between N4 and N3 of C4 and O6 and N1 of I11, respectively. The λY and λR angles of C4s and I11s are approximately 51-55° and 41–49°, respectively, in all four duplexes of the inosine structure. The interstrand C1'–C1' widths of these C4:I11 base pairs in all four inosine duplexes are approximately 10.5-10.8 Å. These C4:I11 base pairs have a shear distance of approximately 0.2-0.5 Å (Supplementary Table S2). Although these parameters do not fulfill all of the aWC criteria, we found a larger λ angle difference in the C:I base pairs (greater than 6) compared to WC (defined as ≤ 4), suggesting that the central C4:I11 base pairs in the inosine substitution structure do not adopt a canonical WC geometry.

Supplementary Note 2. Structural details of the echinomycin binding sites

The overall structures and close-up views of the echinomycin binding sites in C:G-EPX and $_m$C:G-EPX were shown in Figure 4. The two palindromic halves of the C:G-EPX complex containing the d(ACG)$_2$ sequence exhibited a local 2-fold symmetry with an all-atom RMSD of 0.06 Å. Previously reported intermolecular hydrogen bonds enabling ECHI base specificity were also found in the C:G-EPX and $_m$C:G-EPX complexes, including the interactions between N2/N3 of G3 (G13) and CO/NH of ECHI 1 ALA6 (ALA2), N2/N3 of G6 (G10), and CO/NH of ECHI 2 ALA6 (ALA2) (Figure 4C).

The central C4:G11 base pair of the C:G-EPX structure adopts a single *anti–anti* conformation with a hydrogen-bonding pattern similar to that in canonical WC. The λY and λR values for C4 and G11 are 53.3° and 54.4°, respectively. The shear distance is -0.1 Å, while the C1'–C1' distance is 10.5 Å (Supplementary Table S2). These parameter values suggest that the central C4:G11 base pair in the C:G-EPX structure adopts a symmetrical WC conformation similar to that in the C:G-NPX1 structure. On the other hand, the central base pair in $_m$C:G-EPX-1 adopts an *anti–anti* pairing with a hydrogen-bonding pattern similar to that in canonical WC. The λY and λR values for $_m$C4 and G11 are 51.5° and 50.3°, respectively. The shear distance is 0.3 Å and the C1'–C1' width distance is 10.6 Å (Supplementary Table S2). These parameter values suggest that the central $_m$C4:G11 in the $_m$C:G-EPX-1 structure adopts a symmetrical WC conformation similar to the unmethylated structure. On the other hand, the central base pair of the $_m$C:G-EPX-2 structure adopts an *anti-syn* conformation with a hydrogen bonding pattern similar to (w)cWH pairing. The λY and λR are 51.3° and 28°, respectively, the C1'–C1' distance is 10.7 Å,

and the shear distance is 0.2 Å (Supplementary Table S2). The base pair geometry is roughly identical to that of (w)cWH observed in the $_{m}$C:G-NPX-2 structure.

SUPPLEMENTARY FIGURES

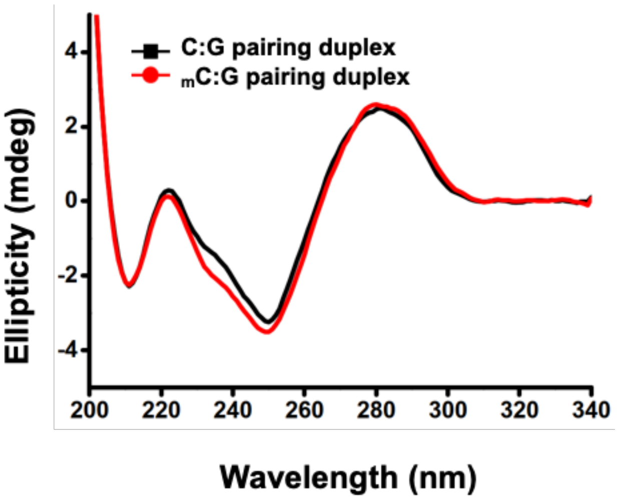

Supplementary Figure S1. CD analysis of unliganded DNA duplexes.

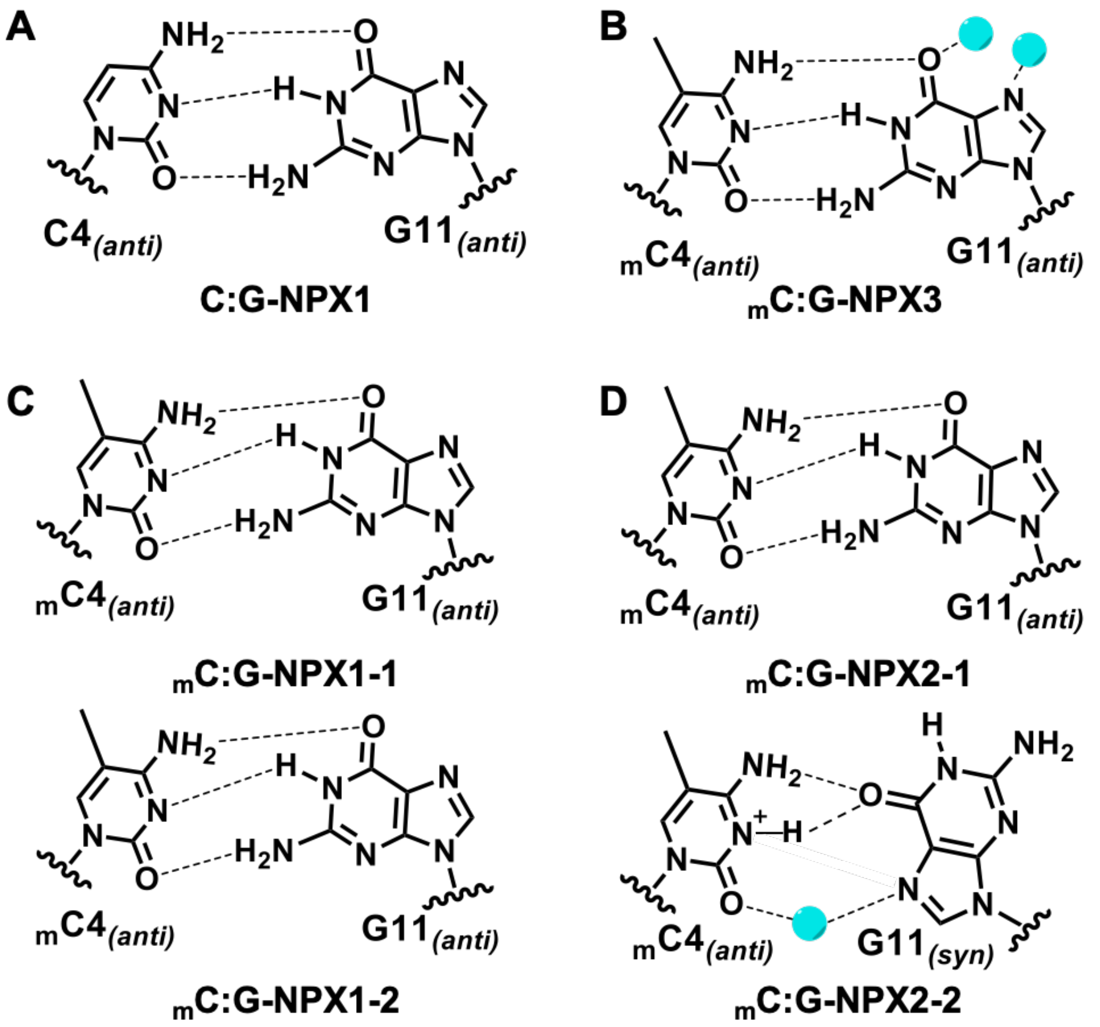

Supplementary Figure S2. (A-D) Schematic diagram of the central C4:G11 base pair of C:G-NPX1 **(A)** and $_{m}$C4:G11 base pairs of $_{m}$C:G-NPX3 **(B)**, $_{m}$C:G-NPX1 **(C)** and $_{m}$C:G-NPX2 **(D)**. Water molecules appear as cyan spheres. Hydrogen bonds are represented by dotted lines.

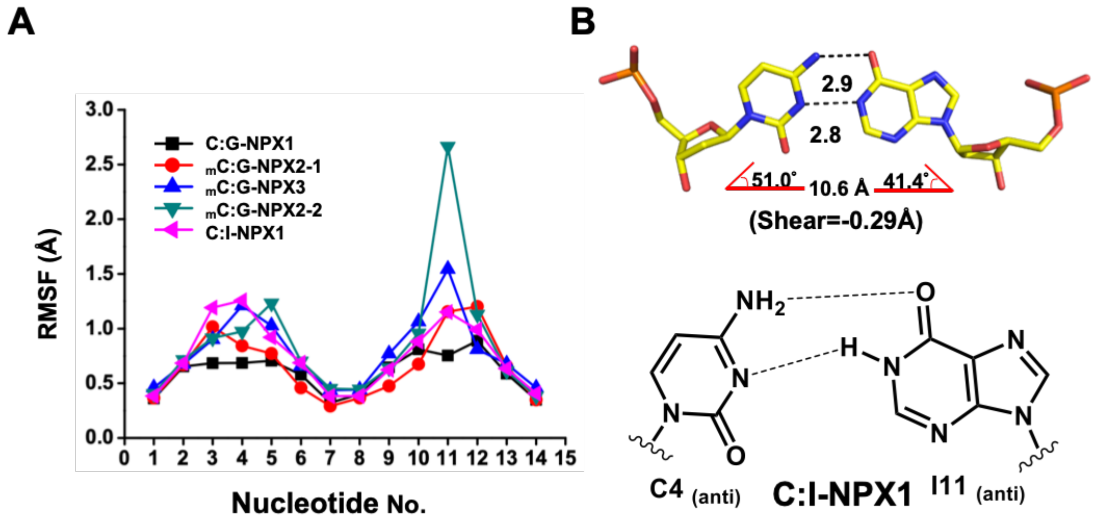

Supplementary Figure S3. (A) RMSF analyses of each base in unliganded DNA duplexes during 1 ns of MD simulations. **(B)** Geometry of the central base pair in the C:I-NPX1 structure. Stick representation (up) and schematic (below) of the central base pair of the C:I-NPX1 structure. Hydrogen bonds are represented by dotted lines, with numbers indicating the distance between two contributing atoms in angstroms (Å). DNA parameters shown at the bottom of the figure include C1'-C1' distance, λ angles and shear.

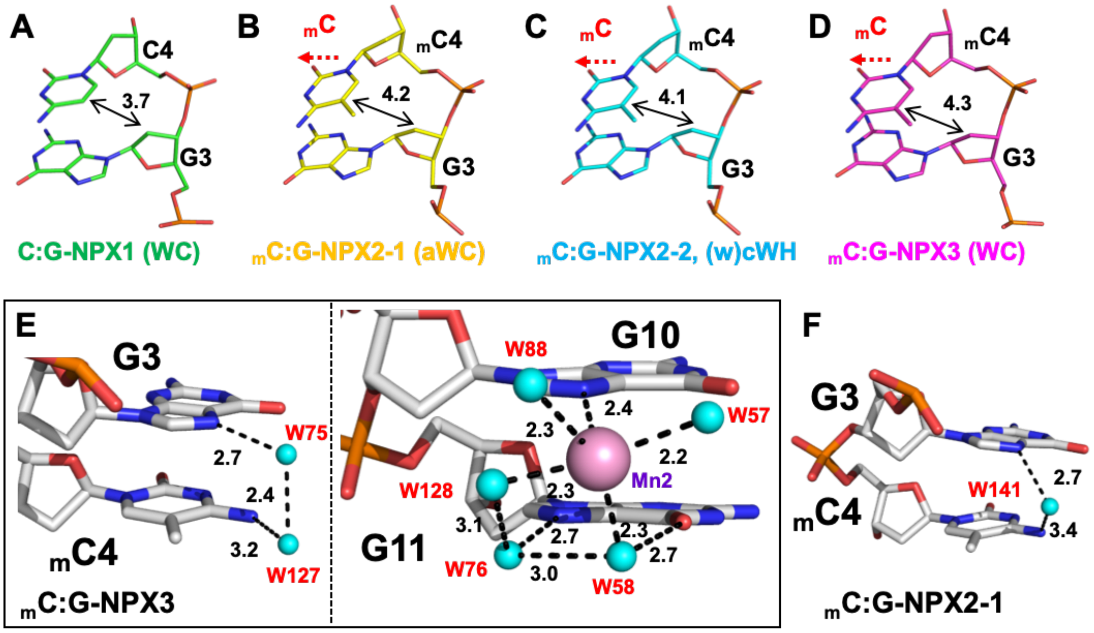

Supplementary Figure S4. (A-D) The distances between the fifth carbon of $_{m}$C4 and the sugar ring of the G3 bases in the indicated duplexes. **(E- F)** Interaction patterns around the central $_{m}$C4:G11 base pair in $_{m}$C:G-NPX3 **(E)** or in $_{m}$C:G-NPX2-1 **(F)**. Metal ions (Mn^{2+}) and water molecules are shown as purple and cyan spheres, respectively. Water-mediated hydrogen bonding and Mn-induced coordination are shown by dashed lines, with numbers indicating the distance between two contributing atoms in angstroms (Å).

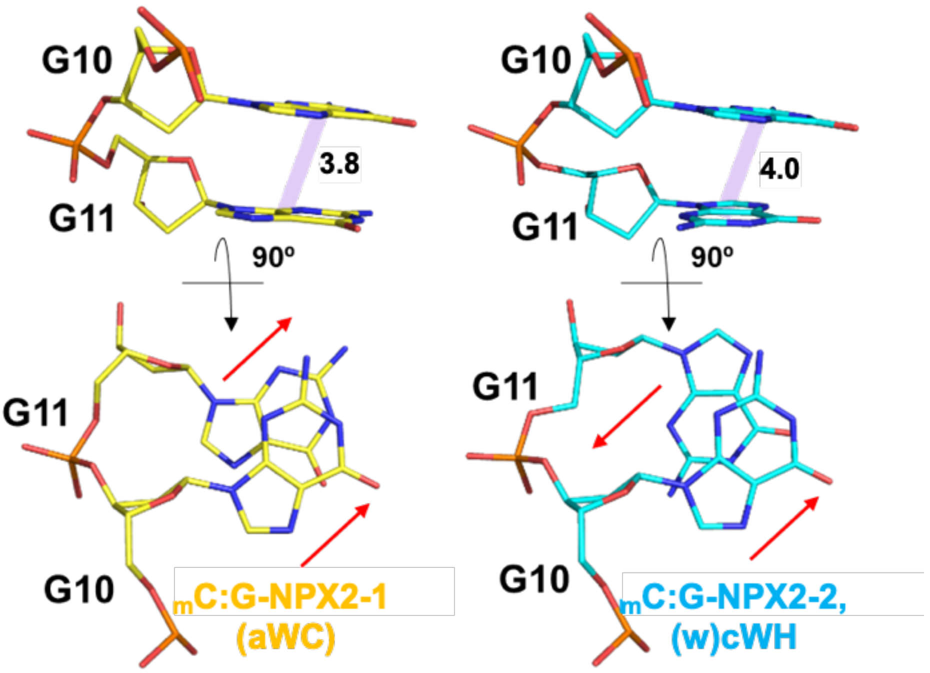

Supplementary Figure S5. Overlap between the π-system of G11 and its 5'-flanking base in $_{m}$C:G-NPX2. Distances are given in Å.

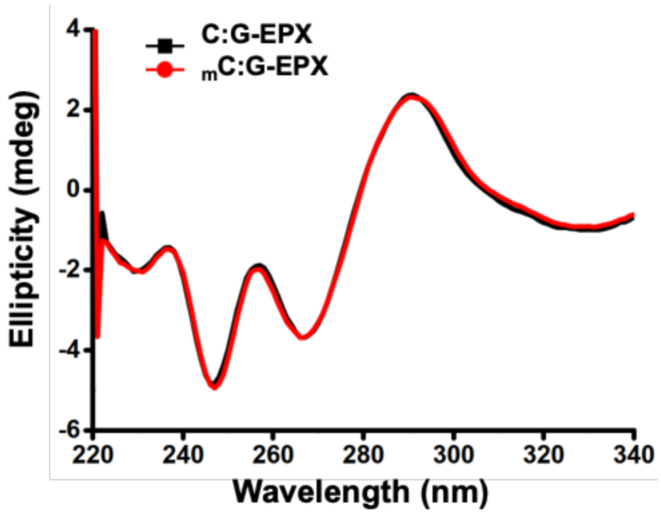

Supplementary Figure S6. CD analysis of C:G-EPX and $_{m}$C:G-EPX complexes.

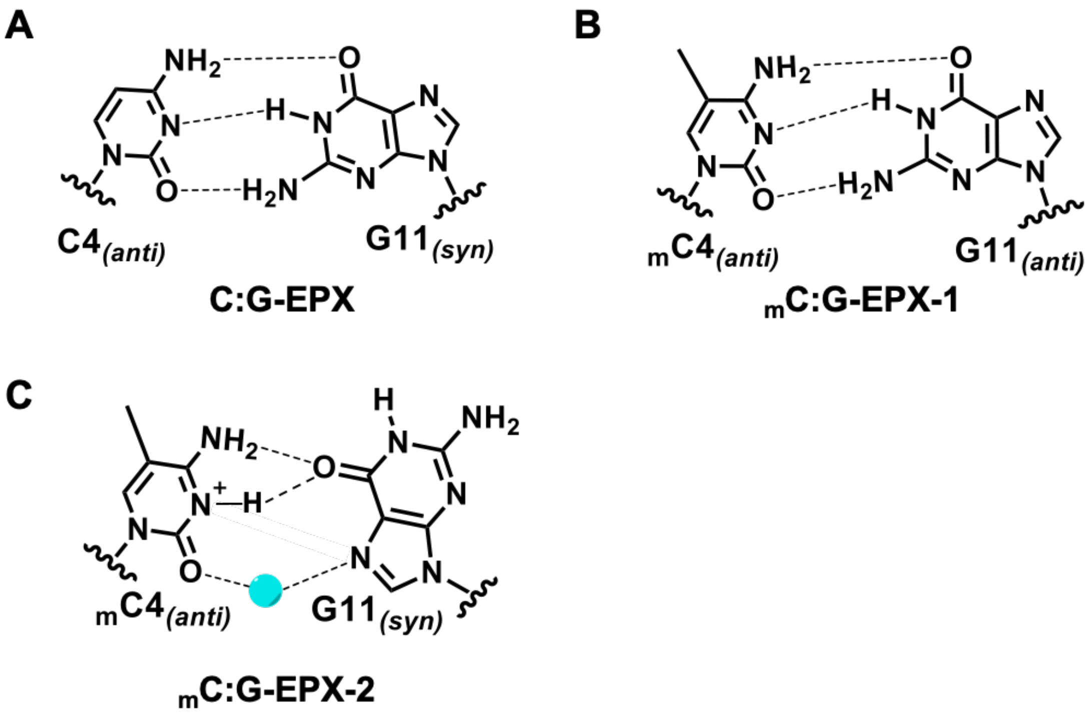

Supplementary Figure S7. (A-C) Schematic diagram of the central C4:G11 base pairs of C:G-EPX **(A)**, $_m$C:G-EPX-1 **(B)** and $_m$C:G-EPX-2 **(C)**. Water molecules appear as cyan spheres. Hydrogen bonds are represented by dotted lines.

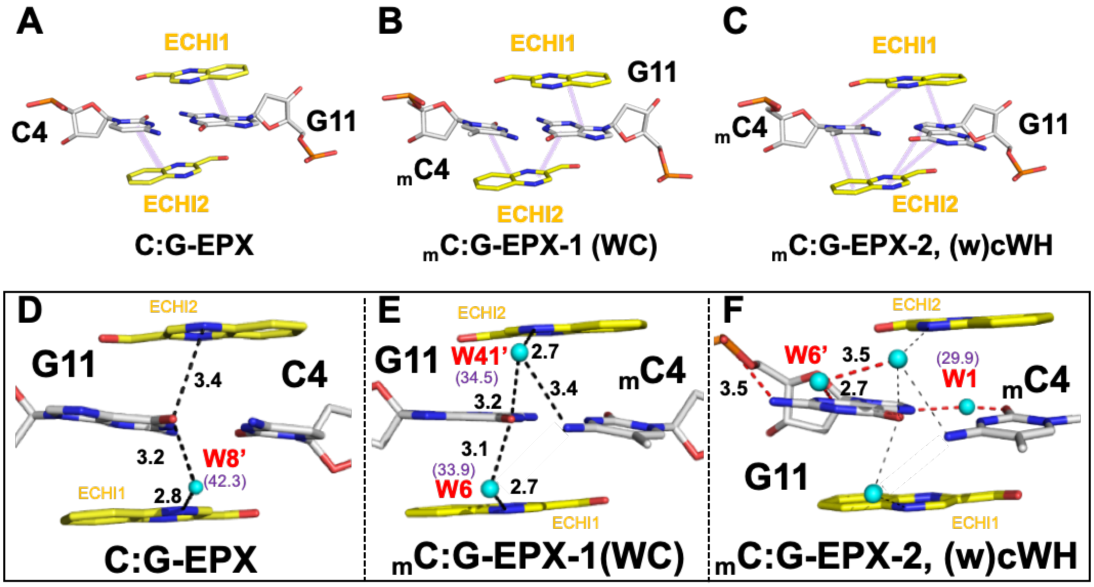

Supplementary Figure S8. (A-C) The π-systems of central base pairs and ECHI in C:G-EPX or $_m$C:G-EPXs. Bases and ECHI are presented as gray and yellow sticks, respectively. Ring-to-ring distances below 5 Å are indicated with purple bars to represent the stacking interactions. **(D-F)** Water-mediated hydrogen bonding helps to stabilize the (w)cWH geometry in the $_m$C:G-EPX-2 structure. Interaction pattern around the central C:G base pair in C:G-EPX **(A)** and around the central $_m$C:G base pairs in mC:G:EPX-1 **(B)** and $_m$C:G:EPX-2 **(C)** are shown. Bases and ECHI are presented as gray and yellow sticks, respectively. Water molecules appear as cyan spheres. Water-mediated hydrogen bonds are shown as dashed black lines. Hydrogen bonds that specifically stabilize the (w)cWH geometry are shown as dashed red lines. Numbers indicate the distance between two contributing atoms in angstroms (Å). B-factor of each water molecules are indicated in purple in brackets.

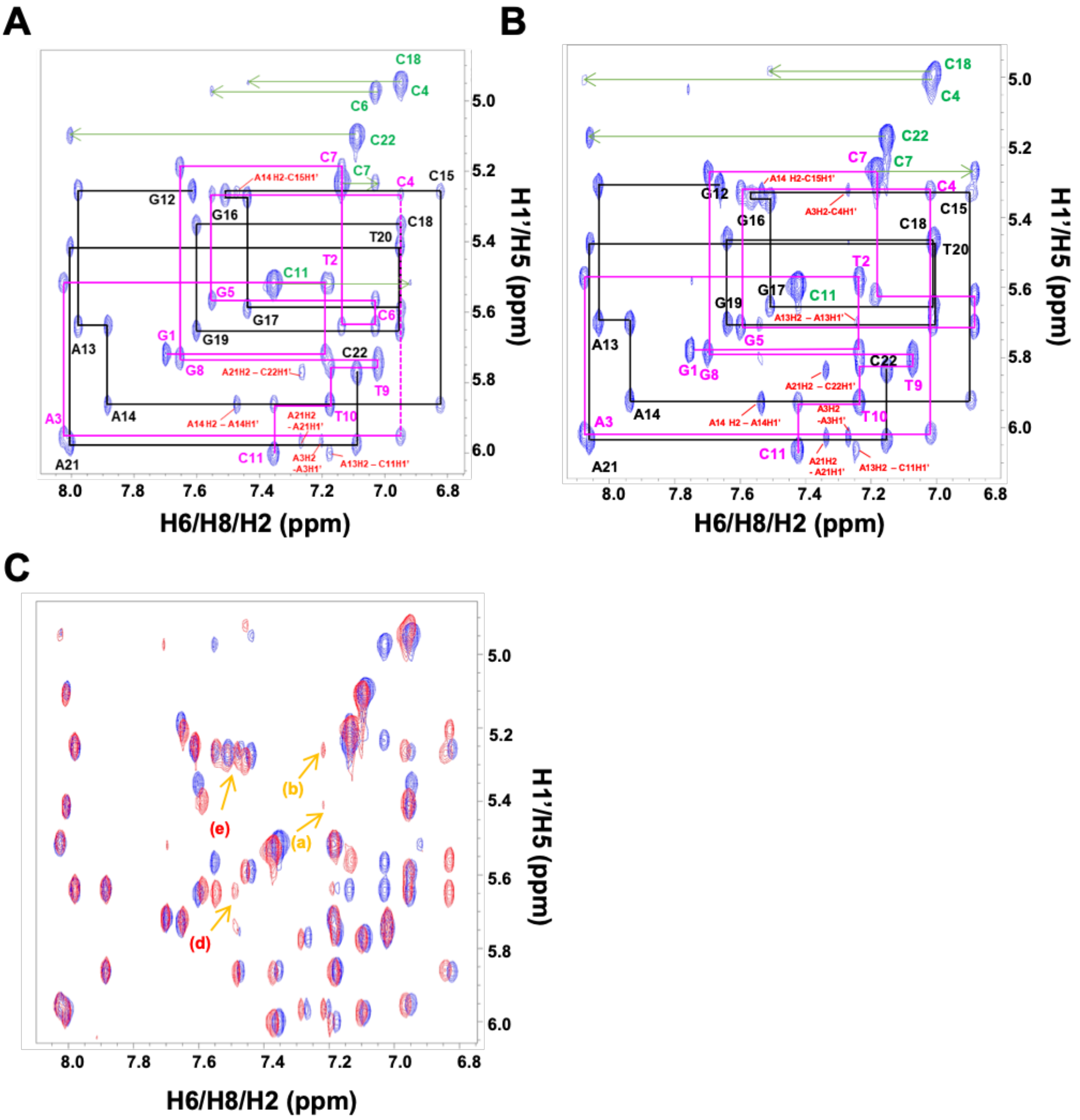

Supplementary Figure S9. The aromatic to H1'/H5 regions of 2D NOESY spectra (τ_m 250 ms) with marked sequential walks for **(A)** LC:G and **(B)** L$_m$C:G. The strong CH5 to CH6 cross-peaks were labeled with green capital letters. The CH5 to H1' cross-peaks were indicated with green arrows. The intra-nucleotide H6/H8 to H1' cross-peaks were labelled with residue numbers and capital letters. Sequential walks of strand 1 and strand 2 were presented with pink and black, respectively. H2 to H1' cross-peaks were presented in red letters. **(C)** A spectral comparison of LC:G (blue) and L$_m$C:G (red) within aromatic to H1'/H5 region. Four additional NOEs observed only in the L$_m$C:G spectrum were marked by arrows with lowercase letters a-b, d-e in brackets, which were further interpreted in Figure 5D.

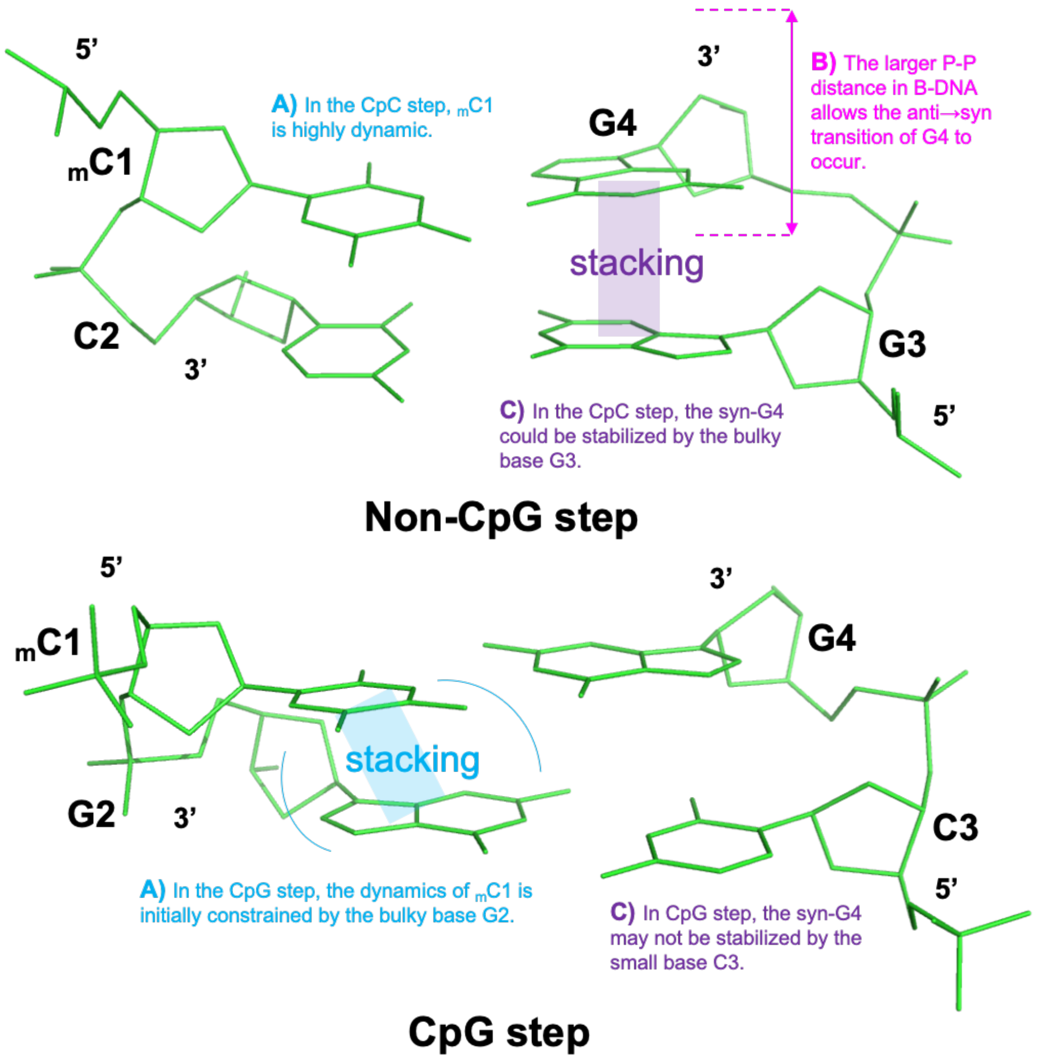

Supplementary Figure S10. The (w)cWH geometry is specific to non-CpG methylated dinucleotide sites, such as CpC or CpT, in B-DNA. **(A)** In the CpC step, the methylated cytosine ($_m$C1) is highly dynamic (upper). In the CpG step, the dynamics of methylated cytosine ($_m$C1) is constrained by its downstream base (G2) (lower). **(B)** In the B-DNA, the larger intra-strand phosphate-to-phosphate (P-P) distance allows the anti→syn transition of the guanine complementary to methylated cytosine (G4) to occur. (C) In the CpC step, the complementary guanine to the methylated cytosine (G4) could be stabilized in a syn-conformation by its upstream bulky base (G3) (upper). In CpG step, the complementary guanine to methylated cytosine (G4) may not be stabilized in a syn-conformation by its its upstream small base (C3) (lower).

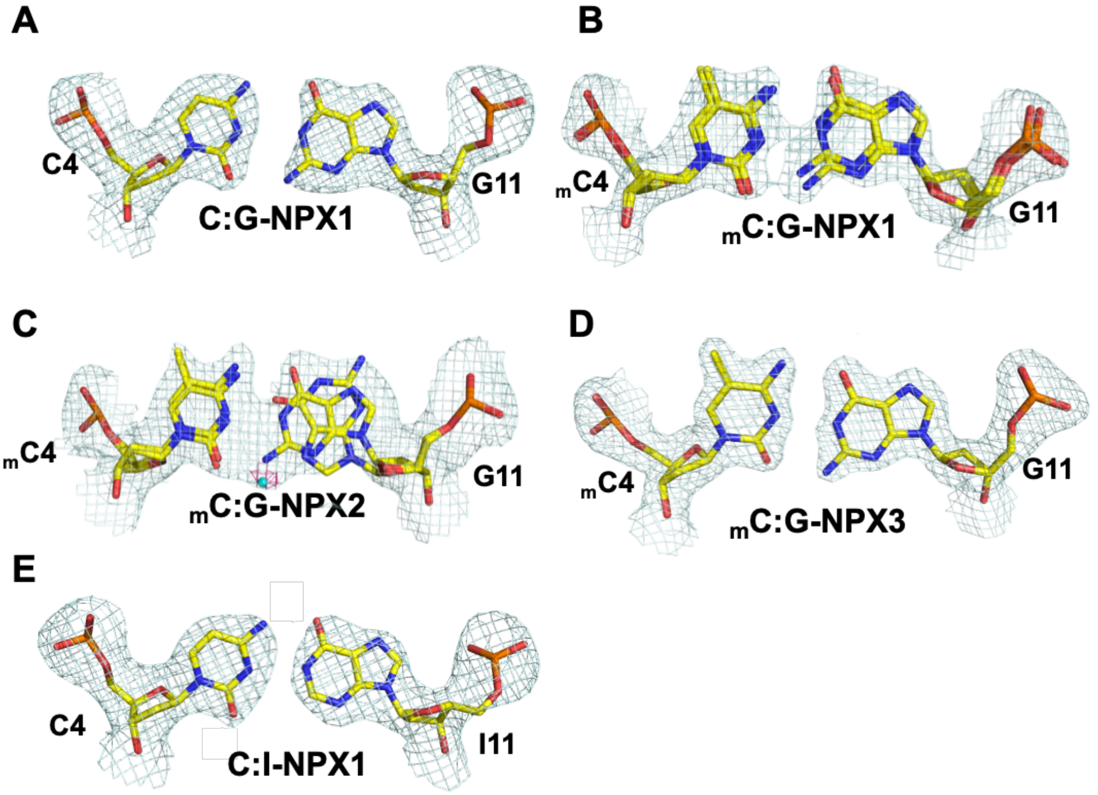

Supplementary Figure S11. Refined electron density maps of central base pairs in C:G-NPX1 **(A)**, $_{m}$C:G-NPX1 **(B)**, $_{m}$C:G-NPX2 **(C)**, mC:G-NPX3 **(D)** and C:I-NPX1 **(E)**. 2Fo-Fc electron density map (contoured at 0.5-1.0 σ, depending on base pair occupancy) and Fo-Fc electron density map (contoured at 2.5 σ) are colored gray and red, respectively. Water molecule is shown as cyan sphere.

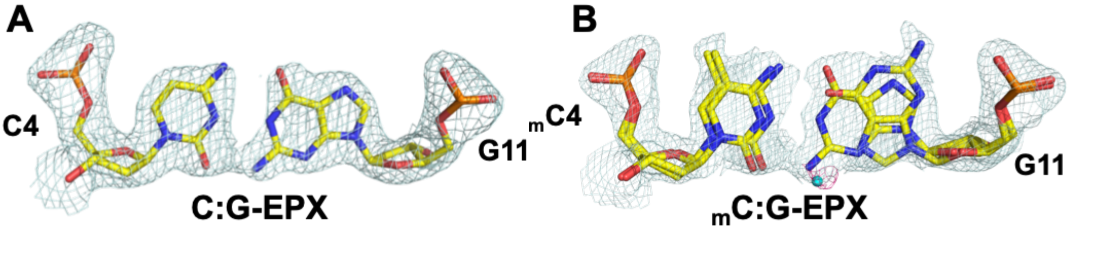

Supplementary Figure S12. Refined electron density maps of central base pairs in C:G-EPX **(A)** and $_{m}$C:G-EPX **(B)**. 2Fo-Fc electron density map (contoured at 0.5-1.0 σ, depending on base pair occupancy) and Fo-Fc electron density map (contoured at 2.5 σ) are colored gray and red, respectively. Water molecules is shown as cyan sphere.

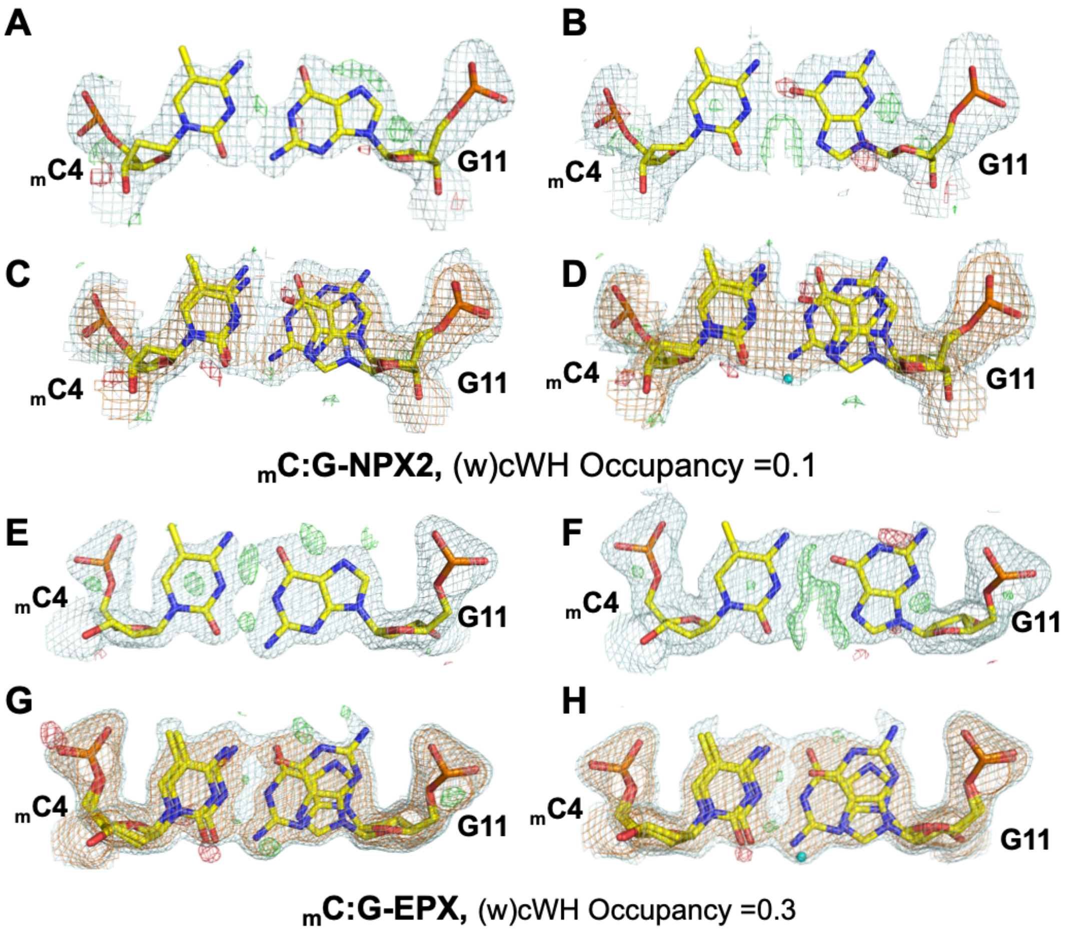

Supplementary Figure S13. 2mFo-DFc and mFo-DFc electron density maps for $_{m}$C:G bps in $_{m}$C:G NPX2s **(A-D)** and $_{m}$C:G-EPXs **(E-H)** generated during refinement, highlighting the assignments of the alternative conformation. Blue and orange meshed regions represent 2mFo-DFc electron density map (contoured at 0.3-1.3 σ, depending on base pair occupancy), while green and red meshed regions indicate mFo-DFc difference densities contoured at 3.5σ and -3.5σ, respectively. Water molecules are shown as cyan spheres.

Supplementary Table S1. Crystallographic data collection and refinement statistics.

**Name**	unliganded C:G pairing duplex	Unliganded $_m$C:G pairing duplex	unliganded CI pairing duplex	C:G-ECHI complex	$_m$C:G-ECHI Complex
**PDB ID**	8XP9	8XPA	8WNB	8XPB	8XP8
**Data collection**					
Space group	$P\ 2_1\ 2_1\ 2_1$	$P2_1$	$P2_12_12_1$	$P3_112$	$P3_112$
Cell dimensions					
a, *b*, *c* (Å)	46.6, 46.7, 75.2	39.4, 46.2, 47.1	46.4, 46.6, 74.7	46.3, 46.3, 48.0	46.0, 46.0, 48.1
a, b, g (°)	-	-, 110.9, -	-	-, -, 120	-, -, 120
Resolution (Å)	22.1 - 2.34 (2.42 - 2.34)	21.50 - 2.00 (2.07 - 2.00)	21.96 -2.49 (2.58 - 2.49)	20.86 - 2.00 (2.07 - 2.00)	16.70 - 1.64 (1.70 - 1.64)
R_{merge}	0.055 (0.624)	0.046 (0.200)	0.042 (0.482)	0.049 (0.483)	0.055 (0.1)
I / s*I*	27.6 (2.3)	22.01 (5.9)	28.1 (3.8)	40.9 (5.9)	33.3 (26.6)
Completeness (%)	99 (96)	100 (99)	100 (97)	100 (100)	100 (100)
Redundancy	5.7	3.6	4.9	10.4	11.1
**Refinement**					
Resolution (Å)	2.34	2.00	2.49	2.00	1.64
No. reflections	7057	10657	5743	4053	7241
R_{work} / R_{free}	0.25/ 0.27	0.22/ 0.25	0.25/ 0.27	0.21/ 0.22	0.23/ 0.24
Number of non-hydrogen atoms	1136	1318	1133	458	566
macromolecules	1124	1212	1120	281	362
Ligand/ion	0	4	0	157	157
solvent	12	102	13	20	47
Average *B*-factors	57.3	43.4	63.5	39.8	33.5
macromolecules					
Ligand/ion	-	30.5	-	37.7	35.9
solvent	55.7	44.1	57.6	44.5	42.7
R.m.s. deviations					
Bond lengths (Å)	0.01	0.03	0.01	0.01	0.02
Bond angles (°)	1.30	2.04	0.86	2.06	2.47

Supplementary Table S2. Comparison of the structural features of the central base pairs in all crystal structures studied.

**Structure name**	**C:G-NPX1**	**C:G-NPX2**	**C:G-NPX3**	**C:G-NPX4**	**${}_{m}$C:G-NPX1-1**	**${}_{m}$C:G-NPX1-2**	**${}_{m}$C:G-NPX2-1**	**${}_{m}$C:G-NPX2-2**	**${}_{m}$C:G-NPX3**	**${}_{m}$C:G-NPX4**	**C:I-NPX1**	**C:I-NPX2**	**C:I-NPX3**	**C:I-NPX4**	**C:G-EPX**	**${}_{m}$C:G-EPX-1**	**${}_{m}$C:G-EPX-2**
**Central base-pair**	C4:G11	C4:G11	C4:G11	C4:G11	${}_{m}$C4:G11	${}_{m}$C4:G11	${}_{m}$C4:G11	${}_{m}$C4:G11	${}_{m}$C4:G11	${}_{m}$C4:G11	C4:I11	C4:I11	C4:I11	C4:I11	C4:G11	${}_{m}$C4:G11	${}_{m}$C4:G11
**Central base-pair geometry**	*anti–anti*	*anti–anti*	*anti–anti*	*anti–anti*	*anti–anti*	*anti–anti*	*anti–anti*	*anti–syn*	*anti–anti*	*anti–anti*	*anti–anti*	*anti–anti*	*anti–anti*	*anti–anti*	*anti–anti*	*anti–anti*	*anti–syn*
**λY / λR**	53.8/54.2	53.3/56.6	53.7/54.5	53.1/53.5	44.6/56.6	44.3/61.7	48.3/59.4	51.6/24.7	55.0/53.5	57.8/54.4	51.0/41.4	52.1/42.0	53.2/43.2	54.9/48.6	53.3/54.4	51.5/50.3	51.3/28
**Central base-pair C1'–C1' distance (Å)**	10.7	10.5	10.6	10.7	10.6	10.6	10.5	10.7	10.7	10.7	10.7	10.7	10.8	10.5	10.5	10.6	10.7
**Shear (Å)**	0.1	0	-0.1	0.2	0.8	1.2	-0.8	-0.2	-0.2	0.2	-0.29	0.22	-0.33	0.48	-0.1	0.3	0.2
**Central base-pair type**	WC	WC	WC	WC	aWC	aWC	aWC	(w)c WH	WC	WC	-	-	-	-	WC	WC	(w)c WH
**Ligand**															V	V	V
**λ angle difference**	0.4	3.3	0.7	0.4	12	17.4	11.1	26.9	1.5	3.4	9.6	10.1	10	6.3	1.1	1.2	23.3

Supplementary Table S3. The geometry of methylated base pairs in the current study and previously reported DNA duplexes.

DNA type	PDBIDs	Sequence[1]	Resolution (Å)	M sites[2]	bp[3] number	bp[3] type	C-C distance (Å)	λ angles (C/G)(º)	P-P distance (C/G)[4](Å)
B-DNA	8XPA	d(ACGMCGT/ACGCCGT)	2.00	CC	M4/G11	(w)c WH	10.5	54.0/24.5	6.4/6.4
	329D	d(ACCGCMGGCGCC)	2.70	CG	M6/G7	WC	10.7	54.5/55	6.7/6.5
					G7/M6	WC	10.5	56.3/58.6	6.5/6.9
	4GJU	d(CGMGAATTCGCG)	1.40		M3/G10	WC	10.7	52.5/54.5	6.6/6.7
					G10/M3	WC	10.9	58.2/59.9	6.4/6.5
	4GLG	d(CGCGAATTMGCG)	1.72		M9/G4	WC	10.6	57.6/54.1	6.5/7.3
	4MKW	d (CGMGAATTCG)	1.22		M3/G22	WC	10.7	54.5/55	6.5/6.7
					G10/M3	WC	10.5	56.3/58.6	6.3/6.5
	6JV5	d (CCAGMGCTGG)	1.40		M5G6	WC	10.8	52.5/53	6.3/6.6
					G6M5	WC	10.8	52.5/53	6.3/6.6
A-DNA	327D	d(GMGMGCGCGC)	1.94		M2/G9	WC	10.6	53.7/58.5	6.0/6.0
					M4/G7	WC	10.7	53.3/56.3	6.0/6.2
					G7/M4	WC	10.7	53.3/56.3	6.0/6.2
					G9/M2	WC	10.6	53.7/58.5	6.0/6.0
	322D	d(CCGGGCCMGG)	2.50		M8/G3	WC	10.8	50.7/56.6	5.4/5.5
	8KG7	d(GTCGGCMCAC/GTGG GCCGAC)	2.20	CC	M7/G4	WC	10.5	58/52.8	6.3/6.4
	1F6E	d(GGCGMC)2 (CPX1)	2.00		M5/G2	WC	10.5	55.4/54.1	5.7/5.5
					G2/M5	WC	10.6	57.8/53.8	5.8/5.9
		d(GGCGMC)2 (CPX2)			M5/G2	WC	10.6	56.1/53.1	5.7/5.7
					G2/M5	WC	10.7	54.8/51.8	5.7/5.6
	1r3z	d(GMGMGCGC)	1.40	CG	M2G7	WC	10.5	55.9/55	6.1/6.1
					M4G5	WC	10.6	56.7/54.7	5.9/6.7
					G5C4	WC	10.6	56.7/54.7	5.9/6.7
					G7M2	WC	10.5	55.9/55	6.1/6.1
	1r41	d(GCGCGMGM)	1.90		M6G3	WC	10.5	53.5/55.3	5.7/6.1
					G3M6	WC	10.5	53.5/55.3	5.7/6.1
E-DNA	1F6I	$d(GGCGMC)_2$	2.20	CC	M5/G2	WC	10.2	57.2/57.3	5.6/5.7
					G2/M5	WC	10.3	60.4/54.9	5.7/5.8

[1]M: methylation cytosine
[2]M sites: methylation sites
[3]bp: base pair
[4]Distance between intra-strand phosphates of the modified base-pair
